# The role of complementary feeding in India’s high child malnutrition rates: findings from a comprehensive analysis of NFHS IV (2015–2016) data

**DOI:** 10.1007/s12571-021-01202-7

**Published:** 2021-09-28

**Authors:** Ivica Petrikova

**Affiliations:** International Relations (Development), Royal Holloway University of London, Egham, UK

**Keywords:** Child malnutrition, India, Infant and young child feeding, Animal-sourced food, ICDS, Poultry ownership

## Abstract

Relative to its economic growth and poverty levels, Indian children suffer from higher levels of malnutrition than children in many other low- and middle-income countries. Research presented in this article examined the links between infant and young child feeding practices among Indian children and their rates of stunting, underweight, wasting, and anaemia, with a particular focus on the types of semisolid complementary food consumed. It did so through a comprehensive analysis of data on more than 57,000 6-to-23-month-old children obtained from the nationally representative National Family Health Survey IV (2015–2016). One of the key findings was that especially feeding children animal-sourced and vitamin-A-rich food was associated with lower malnutrition rates. The study further interrogated whether livestock ownership and participation in the Integrated Child Development Services programme could be supportive of better complementary child feeding and concluded that daily food receipts from the programme and poultry ownership were indeed linked with significantly higher rates of children following the recommended feeding practices as well as with somewhat lower children’s malnutrition rates.

**Supplementary Information:**

The online version contains supplementary material available at 10.1007/s12571-021-01202-7.

## Introduction

On the 2020 Global Hunger Index (GHI, 2020), where three of the four composite indicators relate to child-malnourishment rates, India ranked 94th out of the 107 countries included, well below many economically poorer countries, including neighbouring Nepal (73rd) and Bangladesh (75th). India has managed to reduce child malnourishment in the past few decades, but the rate of progress has been slow and most recent data indicate that it has stalled. From the 22 Indian states^1^ whose results from the National Family Health Survey (NFHS) V (2019–2020) have been released by the time of writing, over the past 5 years only 9 (41%) states experienced a decline in child stunting (too short for age) and only 6 (27%) a decline in child underweight (too light for age). Indian children’s nutrition status is expected to be further detrimentally affected by the ongoing Covid-19 pandemic (e.g. Headey et al., 2020).

Given the negative health, educational, and economic implications of child malnutrition for both the individuals affected and their communities (Dewey & Begum, 2011), further investigations of India’s persistently high rates of child malnutrition are imperative. Infant and young child feeding (IYCF) has been identified, alongside demographic, socio-economic, and water, sanitation, and hygiene (WASH) factors, as a key determinant of children’s nutrition outcomes during their first 1000 days, which are particularly crucial for health outcomes later in life. This article adds to existing knowledge on the topic with comprehensive findings about the relationship between the feeding practices of Indian 6-to-23-month-old children and their nutrition outcomes, based on an econometric analysis of the nationally representative NFHS IV (2015–2016) survey data. The key contribution of this study lies in its detailed exploration of the links between dietary diversity in children’s complementary feeding and their nutrition status, with a particular focus on the children’s intake of animal-sourced food (ASF).

Regarding IYCF, the World Health Organisation (WHO) recommends that all children are breastfed within an hour of birth and breastfed exclusively until they are 6 months old. Thereafter, it advises that children begin receiving semisolid complementary feeds of sufficient frequency and diversity (WHO’s Minimum Acceptable Diet, explained in more detail later). There is a wealth of empirical studies from lower-income countries, including India, on the links between the WHO-recommended IYCF and children’s health and nutrition. Most studies found infants breastfed within an hour of birth, not given pre-lacteal feeds (i.e. non-human milk, water, other liquids etc.), and exclusively breastfed for the first 6 months of their lives to be healthier than their counterparts (Bhutia, 2014; Gupta et al., 2015; Jeyakumar et al., 2019; Mishra et al., 2014; Roche et al., 2017). However, there are also some contradicting findings: Menon et al. (2015) discovered no association between a timely initiation of breastfeeding or exclusive breastfeeding and children’s nutrition and Teshome et al. (2009) found Ethiopian children breastfed after 1 year of age to suffer from higher rates of stunting than non-breastfed children. The authors opined that this was due to a lower nutrient content of the breastfed children’s complementary feeds.

In terms of complementary feeding, initiating it on time has been widely connected with better nutrition outcomes. Children ‘weaned’ late—i.e. given semisolid complementary food later than at 6 months of age—have been frequently found to suffer from higher rates of stunting and underweight than other children (e.g. Bhutia, 2014; Bukusuba et al., 2017; Roche et al., 2017). There is less agreement regarding the ideal weaning age, with some research arguing in favour of the 4-to-6-month age (Shamim et al., 2006; Teshome et al., 2009; Vyas et al., 2014) or even prior to 4 months (Kumar et al., 2006; Padmadas et al., 2002) while others warning about potential negative health consequences of weaning infants too early (Bhutia, 2014; Gupta et al., 2015). Specific to India, most studies concur that the majority of caregivers introduce children to semisolid complementary food too late (e.g. Jayant et al., 2010; Mishra et al., 2014), with one study finding the significantly higher rate of late weaning in India than in Ethiopia a likely partial cause of higher malnutrition prevalence among Indian than Ethiopian children (Petrikova, 2018).

Recent research on IYCF has focused on the importance of *variety* in weaning food, with higher dietary diversity in complementary feeds linked with lower prevalence of stunting, underweight, and wasting (Chandrasekhar et al., 2017, Krasevec et al. 2017, Meshram et al., 2019). However, evidence is emerging that the *type* of complementary food is also important. Predominantly cereal-based weaning in Ethiopia was correlated with higher rates of stunting (Teshome et al., 2009) while the consumption of animal-sourced and iron-rich food (Bukusuba et al., 2017, Krasevec et al. 2017, Roche et al., 2017) with lower stunting rates. This article explores the issue in the Indian context, abundant in vegetarians,^2^ by examining the relationship between different types of weaning food and nutrition outcomes in 6-to-23-month-old children, and finds that particularly the intake of ASF is linked with better outcomes. To obtain more policy-relevant findings, the article then investigates whether the ownership of livestock and participation in the national Integrated Child Development Services (ICDS) programme can help ensure that Indian children adhere to the IYCF habits associated with lower malnutrition rates, including greater consumption of ASF.

Livestock ownership could lead to greater consumption of ASFs even by children from poor families, given the generally high market prices of these items (Headey et al., 2018). Existing research from lower-income countries (e.g. Azzarri et al., 2015; Choudhury & Headey, 2018; Kim et al., 2019) confirms that there is often a positive relationship between owning livestock and children’s consumption of ASF. Nevertheless, Kaur et al.’s (2017) study of 30 African countries indicates that the relationship between livestock ownership and children’s malnutrition rates is heterogeneous, leading in some countries to lower but in other countries to higher prevalence of stunting, mostly due to a greater occurrence of enteric diseases in animal-keeping households.

Participation in the ICDS can also be a potential mechanism of improving children’s feeding practices and nutrition. The ICDS is an Indian national programme, established in 1975, that aims to support better children’s feeding, hygienic, and health practices (UNICEF, 2017). Specific interventions include the delivery of supplementary nutrition and basic health services to children under 6 years old and to pregnant and breastfeeding women and of nutrition and health education to mothers and pre-school education to children. The services are delivered through a network of about 1.3 million *anganwadi* centres (ACWs) across the country (Timsit, 2019). Earlier studies found the ICDS to be unsuccessful in improving children’s nutrition outcomes (e.g. Gragnolati et al., 2006; Kumar et al., 2006; Lokshin et al., 2005), due to a lack of effective nutrition education, parental take-up of only some of the programme’s services, and the programme’s regressive regional and household coverage (Chakrabarti et al., 2019). Some more recent research (Jain, 2015; Kandpal, 2011) concluded that in the twenty-first century, the programme has begun to have a more positive impact on children’s height.^3^ However, a study that examined the effect of the ICDS on beneficiaries’ nutrition noted no significant impact of the programme on calorie or protein intake among children under 3 years old (Mittal & Meenakshi, 2019).

This study finds both livestock ownership and ICDS participation to be at least partially helpful—with children living in districts with a greater ownership of poultry and receiving ICDS benefits consuming more varied diets and more ASF than their counterparts. Accordingly, it concludes that alongside socio-economic factors, better caregivers’ awareness and observation of beneficial IYCF practices, potentially facilitated through strengthening of the ICDS, could play an important role in reducing child-malnutrition rates in India. The rest of the article is organised as follows. Section 2 describes the data and methodology used and Sects. 3 and 4 present the results. The implications of the results and related policy recommendations are discussed in Sect. 5.

## Methodology

### Data and models

The study analyses data on more than 57,000 Indian children between 6 and 23 months of age gathered by NFHS IV (2015–2016), a nationally representative Indian household survey. The lower cut-off age of 6 months was selected due to the WHO recommendation for children from that age onwards to be ‘weaned’; i.e. to receive semisolid food as a supplement to breast milk (WHO, 2005). The upper cut-off point was chosen because the first 2 years of children’s lives are considered the most crucial to ensuring good nutrition and health outcomes in later life (UNICEF, 2017).

The main empirical models examine children’s nutrition outcomes as a function of the children’s IYCF and other characteristics as well as their mothers’, households’, and communal characteristics. The models are estimated using multilevel logistic regressions, because the main dependent variables are all binary and the effects of both individual-level and community-level variables are examined. The first level in the models represents the individual (children or their caretakers) or household, the second level the district, and the third level the Indian state of residence. Other studies of Demographic Health Survey (DHS) datasets such as the NFHS (e.g. Haile et al., 2016, Tassew et al., 2019) noted that the use of multilevel hierarchical models to analyse nested survey data was preferable to the use of standard logistic regression models given that the assumptions of independence among individuals living in the same area and of equal variance across the areas were violated in the case of grouped data (Haile et al., 2016, p. 2). However, as part of sensitivity analysis I estimate the basic models also with regular logistic regressions that control for the state of residence and whose robust standard errors are clustered by districts. Further, given the multitude of outcome variables examined, which raises the likelihood of wrongly rejecting some relationships’ null hypotheses, I conduct multiple hypothesis testing using the code written by Anderson (2008) and from the basic model’s p values calculate variables’ False Discovery Rate (FDR)-sharpened q values.

How livestock ownership influences children’s feeding practices is interrogated through multilevel logistic regressions with appropriately adjusted control variables and how it affects children’s nutrition outcomes by adding the livestock ownership variables to the main models described above. In assessing the impact of ICDS, the study uses propensity score matching (PSM), a quasi-experimental method that is based on the construction of a synthetic suitable control group to the ‘treated’ children on the basis of observable characteristics (e.g. Dixit et al., 2018; Kandpal, 2011; Ravallion, 2001). The output indicators of the ‘constructed’ control group are then subtracted from those of the treated group to determine the size and significance of the treatment’s impact. The specific types of matching employed are the nearest-neighbour and five-nearest-neighbour approaches.

### Variables

#### Dependent variables

The study examines four child malnutrition outcome measures—stunting (too short for age), underweight (too light for age), wasting (too light for height), and suffering from anaemia (lower than normal red-blood-cell count). All four types of malnutrition can be brought about by deficient feeding, but stunting is generally reflective of longer-term while wasting of shorter-term nutrition deprivation. Underweight can be a result of either stunting or wasting whereas anaemia may be caused by iron-deficient diet, alongside frequent diarrhoea and/or intestinal parasites (WHO, 2010).

#### Key independent variables

The basic IYCF indicators investigated include whether a child is weaned (aka, received any semisolid complementary food in the previous 24 h), whether s/he is breastfed, and whether her/his diet in the last 24 h complied with the WHO’s Minimum Acceptable Diet (MAD) guidelines on minimum dietary diversity and minimum frequency of complementary feeding. The WHO (2008, 2018) defines minimum dietary diversity as having consumed, in the 24 h prior to a survey, food from at least four food groups out of the following seven: (1) grains, roots, and tubers; (2) legumes and nuts; (3) dairy products (e.g. yoghurt, cheese); (4) flesh foods (meat, fish, poultry, and organ meats); (5) eggs; (6) vitamin-A rich fruits and vegetables; and (7) other fruits and vegetables. The minimum meal frequency is defined differently for breastfed and non-breastfed children—with breastfed 6-to-8-month-old children needing 2 and 9-to-23-month-old children three non-milk feeds per day, while non-breastfed children between 6 and 23 months of age needing four feeds (along with at least two milk feeds). Abiding by the WHO guidelines on toddler feeding—breastfeeding along with complementary feeding of sufficient diversity and frequency—is expected to reduce the incidence of the four malnutrition measures examined.

Following the initial investigation, the study analyses links between complementary feeding with different food groups and children’s nutrition outcomes using the same models, with the addition of the seven different food types. Further models look specifically at the relationship between the consumption of ASF and children’s malnourishment, including at the effects of consuming more than one source of such food (e.g. both flesh and dairy products or both flesh foods and eggs).^4^

#### Livestock and ICDS

The study analyses four different livestock variables—two household-level, binary ones that enquire if a household owns any cattle or poultry and two communal-level ones measuring the prevalence of cattle and poultry ownership at the district level.^5^ I chose to focus on cattle and poultry due to their relative pervasiveness in India as well as their most common links with the consumption of dairy (cattle), eggs (poultry), and meat (poultry). The rationale for analysing household-level livestock ownership data is that households with livestock may be more likely to consume their home-produced ASF than households without livestock but also potentially live in a more disease-laden environment; the motivation for examining district-level livestock data is that districts with higher livestock ownership may be associated with lower costs and/or more ubiquitous consumption of ASF generally.

Regarding ICDS participation, I examine three measures—whether a child participates in all aspects of ICDS (receives ICDS food at least weekly, regularly attends ICDS preschool, and receives monthly weighing and check-ups) and whether s/he gets food from the programme at least weekly or daily. A link between regular receipts of ICDS food on the one side and good feeding habits and nutrition outcomes on the other can be plausibly anticipated. Meanwhile, previous research (e.g. Gragnolati et al., 2006) suggested that only ‘full participation’ in the ICDS was linked with better nutrition outcomes among children.

#### Control variables

In addition to deficient feeding practices, other child-level factors raising the risk of malnourishment identified in previous studies have included being male, having a low birth weight, and having a short birth interval with a preceding sibling (e.g. Gupta et al., 2015; Jeyakumar et al., 2019; Mishra et al., 2014). Older, more nourished, more educated mothers with fewer children, from wealthier households, were found to be less likely to have malnourished children (Bharati et al., 2018; Bhutia, 2014; Menon et al., 2018; Mishra et al., 2014, Patel et al., 2012). Other variables put forward as potentially important have included households’ religion, caste, type of residential area, and the season when data were collected^6^ (Bharati et al., 2018; Bhutia, 2014; Kumar et al., 2006; Roba et al., 2019). Finally, WASH factors have been increasingly linked with children’s nutrition outcomes, with Indian children living in households with improved toilets and/or in districts with greater prevalence of such households found to be proportionally less malnourished (e.g. Meshram et al., 2019; Spears et al., 2013).

In line with existing research, this study controls for the following child characteristics: the child’s gender, age group (6–11, 12–17, or 18–23 months old), birth order, and birth interval with preceding sibling and whether s/he was born preterm, received any pre-lacteal feeds, was breastfed within one hour of birth, and suffered from diarrhoea in the preceding 2 weeks. The models also contain maternal (age at birth, underweight, and years of education completed) and household characteristics (household size, female-headed household, number of under-five children, caste, religion, wealth index quintile,^7^ and owning a private improved toilet). Communal-level control variables include district-level prevalence of private improved toilets and whether the district is coastal or inland and urban or rural. Most of the control variables were also used in calculating the propensity scores to estimate the effects of the ICDS; this is described in more detail in the PSM document in the Appendix.

## Summary statistics

Table 1 displays summary statistics of all the variables used. A sampling weight was used in the estimations, to ensure that the summary statistics were nationally representative (DHS, 2018, 1.29–1.30). The first part of the table shows that the proportions of 6-to-23-month-old Indian children analysed in the study suffering from malnutrition in 2015–2016 were high: 36% were stunted, 34% underweight, 24% wasted, and 58% anaemic.

**Table 1:** Descriptive statistics of the variables used

Variable	N	Mean	Std. Dev	Min	Max
Nutrition outcomes					
Stunted	57,021	0.36	0.48	0	1
Underweight	57,021	0.34	0.47	0	1
Wasted	57,021	0.24	0.43	0	1
Anaemic	57,021	0.58	0.50	0	1
Infant & young child feeding					
Weaned (fed semisolid food in last 24 h)	57,021	0.77	0.42	0	1
Minimum dietary diversity	57,021	0.20	0.40	0	1
Minimum dietary frequency	57,021	0.29	0.50	0	1
Breastfed (currently)	57,021	0.87	0.34	0	1
Consumed in last 24 h					
Grains, roots, and tubers	57,021	0.71	0.45	0	1
Legumes and nuts	57,021	0.13	0.34	0	1
Dairy products	57,021	0.28	0.45	0	1
Flesh foods	57,021	0.10	0.30	0	1
Eggs	57,021	0.14	0.35	0	1
Vit.A-rich fruits and vegetables	57,021	0.40	0.49	0	1
Other fruits and vegetables	57,021	0.35	0.48	0	1
Any animal-source foods	57,021	0.36	0.48	0	1
Livestock ownership and ICDS					
Cattle (cow, bull, buffalo)	57,021	0.39	0.49	0	1
Poultry (chicken, duck, other)	57,021	0.13	0.34	0	1
Cattle—district level	57,021	0.39	0.18	0	0.88
Poultry—district level	57,021	0.13	0.15	0	0.97
Daily food from ICDS	57,021	0.20	0.40	0	1
Weekly (at least) food from ICDS	57,021	0.34	0.47	0	1
Full participation in ICDS	57,021	0.08	0.27	0	1
Child characteristics					
Female child	57,021	0.48	0.50	0	1
Age bracket					
6–11 months		0.35			
12–17 months		0.34			
18–23 months		0.32			
Preterm	57,021	0.06	0.24	0	1
Any prelacteal feed	57,021	0.21	0.41	0	1
Breastfed within 1 h of birth	57,021	0.69	0.46	0	1
Had diarrhoea in the last 2 weeks	57,021	0.15	0.36	0	1
Birth order	57,021	2.21	1.38	1	15
Birth interval	57,021	24.25	25.75	0	275
Maternal characteristics					
Mother’s age at birth	57,021	25.03	4.66	14	49
Mother undernourished	57,021	0.21	0.41	0	1
Mother’s years of education	57,021	6.67	5.18	0	20
Household characteristics					
HH size	57,021	6.45	2.91	2	41
Female head of HH	57,021	0.12	0.33	0	1
No. of children under 5	57,021	1.80	0.88	0	9
Caste	57,021				
Scheduled caste		22.66			
Scheduled tribe		20.98			
Other backward caste		45.80			
Upper caste		19.60			
Other		0.95			
Religion	57,021				
Hindu		80.62			
Muslim		14.55			
Other		4.83			
Wealth index quintile	57,021				
Lowest		23.39			
Lower middle		20.39			
Middle		19.22			
Upper middle		19.10			
Highest		17.90			
Season of interview	57,021				
Winter		11.14			
Summer		57.71			
Monsoon		30.09			
Autumn		1.05			
Private improved toilet	57,021	0.40	0.49	0	1
Communal characteristics					
District toilet prevalence	57,021	0.39	0.21	0.04	1
Coastal district	57,021	0.13	0.33	0	1
Urban area	57,021	0.27	0.44	0	1

Looking at children’s dietary habits, 77% of the children examined were fed some semisolid complementary food in the 24 h prior to the survey, with the proportion higher in older age categories: 6–11 months—57%, 12–17 months—84%, and 18–23 months—90%. The diet of only 29% of children complied with the WHO guidelines on minimum feeding frequency and of only 20% on minimum dietary diversity. In contrast, the rates of breastfeeding were very high, with 87% of the children breastfed. The most consumed food group were grains, roots, and tubers, followed by vitamin-A rich fruits and vegetables and other fruits and vegetables. 36% of the sampled children were fed some ASF the day before the survey. The least commonly consumed food group was meat (flesh foods), with only 10% children having eaten any the day prior to the survey.

Regarding the livestock and ICDS variables, 39% of the sample households owned some type of cattle (cow, bull or buffalo) and 13% some poultry (chickens, ducks or other). The district-level prevalence of ownership varied between 0 and 88% for cattle and between 0 and 97% for poultry. In terms of the ICDS benefits, 20% of children in the sample received daily food rations from the ICDS and 34% food at least weekly. Only 8% children participated in all the measured parts of the programme—i.e. receipts of food rations at least weekly and of medical check-ups and weigh-ins at least monthly, with regular attendance of the ICDS preschool.

Turning to summary statistics of the control variables, slightly fewer than half of the sample were female children. 6% had been born prematurely, 21% had been given a pre-lacteal feed, 69% had been breastfed within one hour of birth, and 15% had suffered from diarrhoea in the two weeks before the survey. The average child in the survey had been born second in the family, with the older sibling approximately 2 years older.

From maternal and household characteristics, the average age of the child’s mother at birth was 25 years and 21% mothers in the sample were undernourished. The average mother completed close to 7 years of education but 28% of the mothers surveyed had received no formal education at all. The average household size was six, with 12% households female-headed. About 46% households were from ‘other backward castes’, 23% from scheduled castes, 21% from scheduled tribes, and the rest from upper and other castes. 81% households were Hindu, 15% Muslim, and 4% of other religious persuasion including Christianity, Sikhism, and Buddhism. 40% households had access to a private improved toilet. Most households were interviewed during the summer, followed by monsoon and winter. 13% of the households examined lived in coastal districts and 27% in urban areas.

## Results

### Baseline models: children’s IYCF and their nutrition outcomes

The baseline models presented in Table 2 indicate that from the four basic IYCF factors examined, weaning (= semisolid complementary feeding) and minimum dietary diversity both have a consistently statistically significant positive relationship with lower malnutrition occurrence among 6-to-23-month-old Indian children, particularly with lower rates of stunting and underweight, and in the case of weaning also with reduced occurrence of wasting. These findings are robust to different estimation models (Table 7 in the Appendix) as well as to multiple hypothesis testing through the calculation of FDR-sharpened q values (Table 8 in the Appendix).^8^ In contrast, neither minimum feeding frequency nor breastfeeding are associated with lower malnutrition rates.

**Table 2:** Links between IYCF and nutrition outcomes in 6-to-23-month-old Indian children

Nutrition outcome	Stunted		Underweight		Wasted		Anaemic	
Model	(1) full	(2) no soc.-econ	(3) full	(4) no soc.-econ	(5) full	(6) no soc.-econ	(7) full	(8) no soc.-econ
Infant & young child feeding								
Weaned (fed semisolid food)	0.919*	0.900**	0.892***	0.876***	0.874***	0.874***	0.993	0.990
	0.035	0.034	0.024	0.023	0.025	0.024	0.024	0.023
Minimum dietary diversity	0.912*	0.865***	0.902***	0.872***	0.956	0.942*	0.966	0.954*
	0.035	0.033	0.024	0.023	0.028	0.027	0.022	0.021
Minimum dietary frequency	1.014	1.014	0.987	0.976	1.017	1.007	1.016	1.018
	0.034	0.034	0.023	0.022	0.025	0.025	0.021	0.020
Breastfed	1.133**	1.208***	1.210***	1.263***	1.126***	1.148***	0.934*	0.950˚
	0.052	0.055	0.039	0.040	0.040	0.040	0.026	0.026
Child characteristics								
Female child	0.785***	0.790***	0.782***	0.790***	0.878***	0.878***	0.983	0.988
	0.022	0.022	0.015	0.015	0.018	0.018	0.017	0.016
Age (comp. to 6–11 months)								
12–17 months	2.374***	2.374***	1.316***	1.330***	0.901***	0.897***	0.999	1.001
	0.089	0.087	0.033	0.032	0.023	0.023	0.022	0.021
18–23 months	3.672***	3.710***	1.721***	1.742***	0.773***	0.772***	1.008	1.015
	0.142	0.141	0.044	0.044	0.021	0.021	0.023	0.023
Preterm	1.248***	1.270***	1.175***	1.185***	1.063	1.065	1.027	1.021
	0.071	0.071	0.047	0.046	0.045	0.045	0.037	0.036
Any prelacteal feed	0.996	0.979	0.969	0.945*	0.989	0.977	1.020	1.015
	0.036	0.036	0.026	0.025	0.028	0.028	0.024	0.024
Breastfed within 1 h of birth	1.066˚	1.050	1.014	1.017	1.008	1.011	0.954*	0.963˚
	0.035	0.034	0.024	0.023	0.025	0.025	0.020	0.020
Had diarrhoea in the last 2 weeks	1.011	1.021	1.136***	1.137***	1.121***	1.124***	1.030	1.030
	0.039	0.039	0.031	0.030	0.032	0.032	0.026	0.025
Birth order	1.063***	1.161***	1.071***	1.154***	1.036***	1.067***	1.021*	1.046***
	0.015	0.015	0.011	0.011	0.011	0.011	0.009	0.009
Birth interval	0.997***	0.998***	0.997***	0.997***	0.998***	0.998***	1.000	1.000
	0.001	0.001	0.000	0.000	0.000	0.000	0.000	0.000
Maternal characteristics								
Mother’s age at birth	0.993˚	0.982***	0.985***	0.976***	1.000	0.997	0.993**	0.990***
	0.004	0.004	0.003	0.003	0.003	0.003	0.002	0.002
Mother undernourished	1.367***	1.458***	1.904***	2.032***	1.515***	1.562***	1.197***	1.231***
	0.044	0.047	0.044	0.046	0.037	0.038	0.027	0.027
Mother’s education in years	0.959***		0.963***		0.986***		0.986***	
	0.003		0.002		0.003		0.002	
Household characteristics								
HH size	0.989˚	0.979***	0.988**	0.975***	0.994	0.988**	1.010**	1.003
	0.006	0.005	0.004	0.004	0.004	0.004	0.004	0.004
Female head of HH	0.995	1.020	1.006	1.017	0.963	0.973	0.992	0.987
	0.042	0.042	0.030	0.030	0.031	0.031˚	0.026	0.026
Number of children under 5 in HH	1.106***	1.130***	1.074***	1.091***	1.021	1.026	1.008	1.014
	0.022	0.022	0.015	0.015	0.015	0.015	0.013	0.013
Caste (comp. to scheduled caste)								
Scheduled tribe	0.897*		0.962		1.125**		1.180***	
	0.044		0.034		0.043		0.040	
Other backward caste	0.867***		0.898***		0.992		0.904***	
	0.033		0.024		0.028		0.022	
Upper caste	0.671***		0.752***		0.913*		0.873***	
	0.035		0.026		0.034		0.026	
Religion (comp. to Hindu)								
Muslim	1.054		0.969		0.963		0.862***	
	0.047		0.031		0.034		0.025	
Other (Sikh, Christian, Buddhist, Traditional…)	0.974		0.749***		0.748***		0.807***	
	0.080		0.038		0.040		0.035	
Wealth quintile (comp. to lowest)								
Lower middle	0.865***		0.893***		0.943˚		0.976	
	0.034		0.025		0.029		0.026	
Middle	0.816***		0.780***		0.893**		0.974	
	0.037		0.025		0.031		0.029	
Upper middle	0.725***		0.689***		0.840***		0.930*	
	0.035		0.025		0.033		0.030	
Highest	0.639***		0.575***		0.763***		0.887**	
	0.041		0.025		0.036		0.034	
Season of interview (comp. to winter)								
Summer	1.002	0.973	1.204***	1.166***	1.224***	1.215***	1.189***	1.197***
	0.040	0.038	0.054	0.052	0.059	0.058	0.051	0.050
Monsoon	1.145**	1.127**	1.460***	1.395***	1.426***	1.393***	1.295***	1.306***
	0.050	0.048	0.074	0.070	0.077	0.076	0.066	0.065
Autumn	1.142	1.187*	1.100	1.114	1.191	1.256*	1.102	1.135
	0.107	0.102	0.113	0.110	0.133	0.136	0.115	0.110
Private improved toilet	0.870***	0.686***	0.876***	0.676***	0.909***	0.802***	0.916***	0.826***
	0.033	0.023	0.022	0.015	0.024	0.019	0.020	0.016
Communal characteristics								
District toilet prevalence	0.932	0.612***	0.450***	0.304***	0.596***	0.503***	0.644***	0.566***
	0.087	0.050	0.040	0.026	0.056	0.045	0.063	0.051
Urban	1.011		1.037		1.038		0.954*	
	0.043		0.028		0.030		0.022	
Coastal	0.835**		1.013		1.202**		1.063	
	0.057		0.065		0.081		0.075	
ICC	0.102	0.104	0.045	0.041	0.043	0.040	0.062	0.059

All regressions were estimated using multilevel logistic regressions with the first level individual/household, second level the district, and third level the Indian state. The numbers next to the variables are adjusted odd ratios (AOR), below are standard errors. The N for all regressions is 57,021. The interclass correlation coefficient (ICC) shows that between 4 and 10% variation in children’s nutrition outcomes is due to community-level variables

***p < 0.001; **p < 0.01; *p < .05; °*p* < 0.1

Theoretically, one could expect that many differences in IYCF feeding relate to households’ different socio-economic circumstances. Poorer households may be unable to afford to provide a sufficiently diverse weaning diet while less educated mothers might be less aware of the existing recommendations on children’s ideal weaning age and diet composition. Table 2 validates this expectation to some extent—the full models, which control for all socio-economic variables, do show lower significance on most of the nutrition outcomes than the models excluding socio-economic control variables. Nevertheless, in most cases, except for the relationship between minimum dietary diversity on the one side and wasting and anaemia on the other, the significant associations remain even in the full models.

**Fig. 1:**
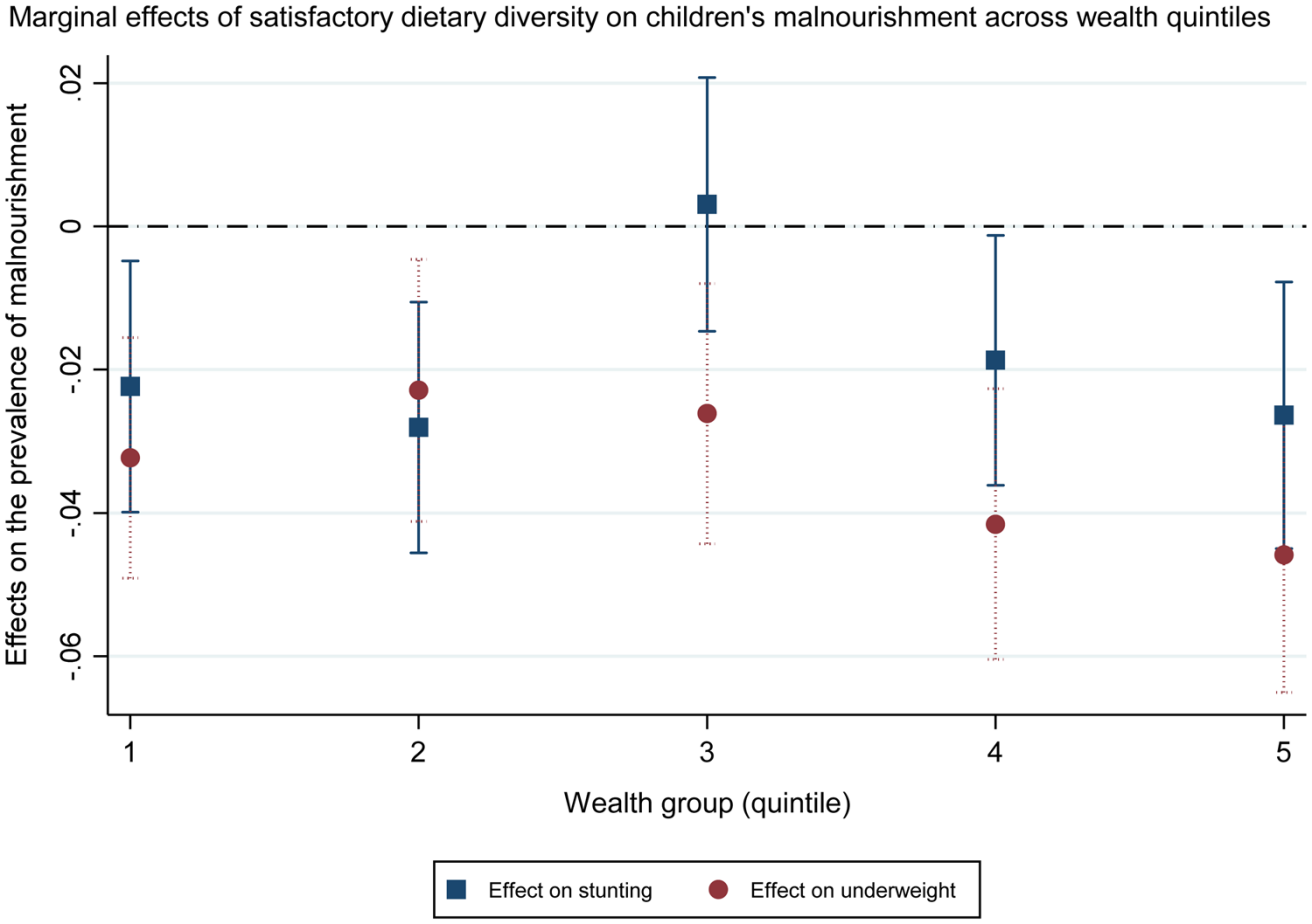
Links between minimum dietary diversity and children’s stunting and underweight across different wealth groups. Marginal effects calculated from regressions akin to models (1) and (3) in Table 2, with the addition of an interaction term between minimum dietary diversity and households’ wealth quintile

An examination of the interaction between minimum dietary diversity of complementary feeds and households’ wealth quintile also demonstrates that it is not only in the poorest Indian households where poor IYCF habits occur and correlate with children’s nutrition outcomes. Figure 1 shows that feeding Indian toddlers the minimum diverse diet is associated with lower prevalence of stunting and underweight in all wealth quintiles except for the middle one, with the association strongest not only in the two poorest but also in the richest quintile.

**Fig. 2:**
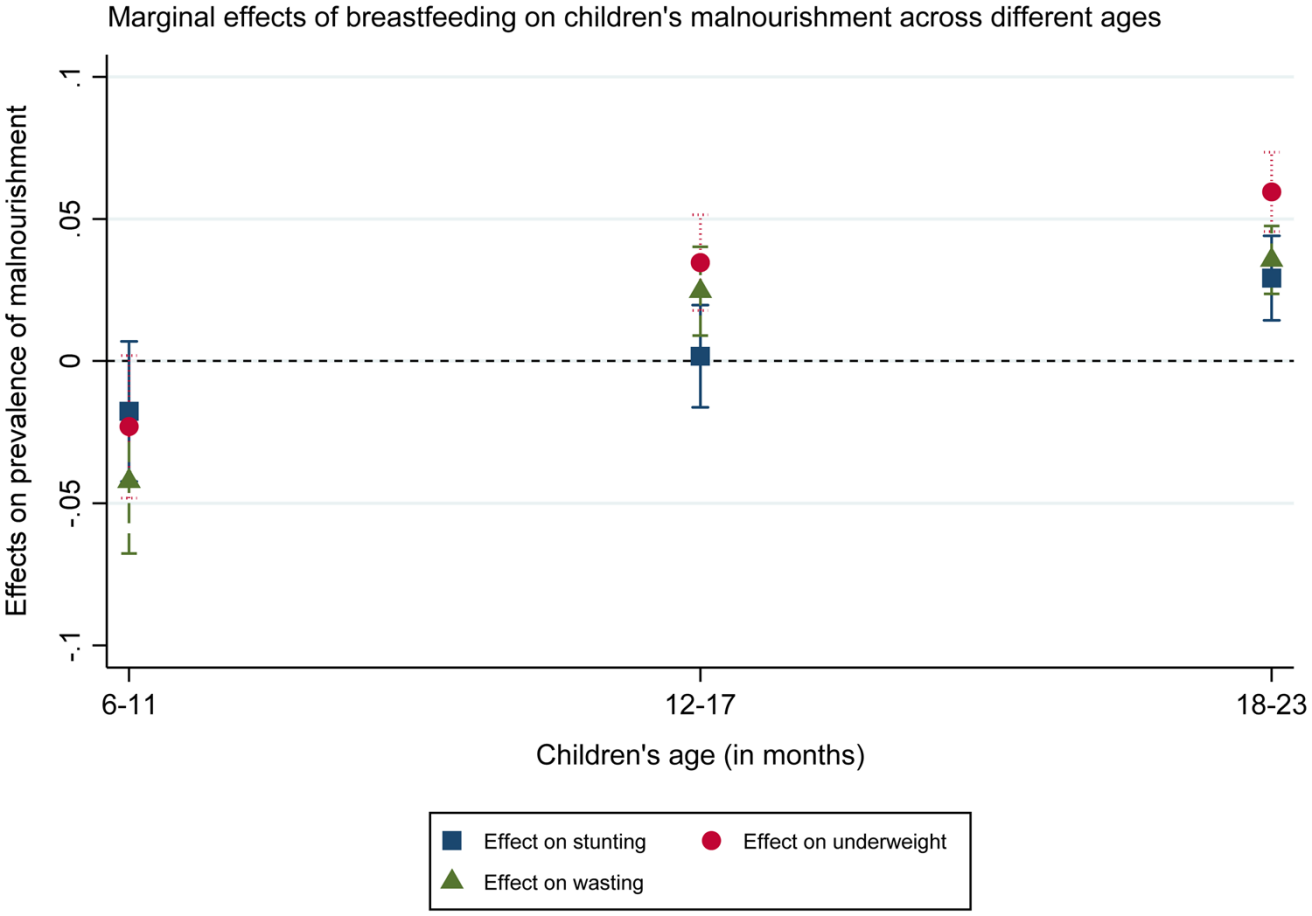
Links between breastfeeding and children’s nutrition outcomes by age group. Marginal effects calculated from regressions akin to models (1), (3), and (5) in Table 2, with the addition of an interaction term between breastfeeding and children’s age group

As noted already, Table 2 does not show minimum dietary frequency and breastfeeding, unlike weaning and minimum dietary diversity, to be significantly linked with lower malnutrition prevalence. The relationship between feeding frequency and nutrition outcomes is largely statistically insignificant while breastfeeding is, at first glance counter-intuitively, associated with higher odds of child stunting, underweight as well as wasting. A closer look into the effects of breastfeeding on stunting, underweight, and wasting by age groups, displayed in Fig. 2, shows, however, that the positive link is only significant for underweight and wasting in children older than 12 months and for stunting only for children older than 18 months.^9^ Previous research has suggested that such findings could be due to a lower nutrient quality of breastfed children’s complementary feeds (Teshome et al., 2009) or due to reverse causality—i.e. where women with small/and or thin children continue breastfeeding longer to try to support their children’s growth (Krasevec et al., 2017). In other words, it is not breastfeeding that leads to children’s negative nutrition outcomes in that case but the poor nutrition status that inspires prolonged breastfeeding.

### Links between different types of complementary food and children’s nutrition outcomes

Table 3, using the same estimation models as Table 2 with the addition of weaning food groups,^10^ suggests that particularly feeding children ASF and vitamin-A-rich fruits and vegetables may reduce their odds of malnourishment. Feeding children flesh and dairy products is associated with lower risks of stunting, underweight, and anaemia and eggs with a lower risk of stunting. Meanwhile, the consumption of vitamin-A-rich plants is linked with a significantly lower prevalence of underweight and wasting and of other fruits and vegetables with a lower prevalence of anaemia.

**Table 3:** Links between different complementary food groups and children’s nutrition outcomes

	Stunted	Underweight	Wasted	Anaemic
Flesh foods	0.909**	0.881**	0.988	0.918*
	0.033	0.035	0.043	0.031
Eggs	0.966˚	1.010	1.043	0.991
	0.027	0.037	0.042	0.031
Dairy foods	0.943*	0.936***	0.104	0.950**
	0.023	0.024	0.028	0.021
Legumes and nuts	1.026	0.979	0.985	1.038
	0.031	0.032	0.034	0.029
Grains, roots, and tubers	0.957	0.973	0.979	0.943
	0.038	0.041	0.044	0.034
Vitamin-A rich plants	1.001	0.932**	0.942**	1.028
	0.023	0.023	0.024	0.022
Other plants/foods	0.993	0.995	1.001	0.961˚
	0.023	0.024	0.026	0.021
Any animal-sourced food	0.895***	0.897***	0.995	0.926***
	0.020	0.021	0.025	0.019
Flesh and eggs	0.934***	0.919***	0.968	0.954**
	0.017	0.018	0.020	0.016
Flesh and dairy	0.917**	0.831***	0.959	0.953
	0.038	0.039	0.048	0.036
Eggs and dairy	0.972	0.899**	0.960	0.946˚
	0.036	0.037	0.043	0.031
Flesh, eggs, and dairy	0.907*	0.839***	0.946	0.934˚
	0.041	0.043	0.052	0.032

The numbers next to the variables are AORs, below are standard errors. The N for all regressions is 57,021

The coefficients are derived from regressions akin to (1), (3), (5), and (7) in Table 2, with the addition of the specific variables (or their interaction) displayed in this table and the subtraction of satisfactory dietary diversity and weaned

***p < 0.001; **p < 0.01; *p < .05; °*p* < 0.1

Results of a closer examination of the relationships between eating different types of ASF and children’s nutrition outcomes follow in the lower part of Table 3. The relationship between whether the toddlers consumed any source of animal food in the 24 h prior to the survey and their nutrition status was interrogated, along with the relationships between different combinations of animal-sourced complementary foods and their nutrition outcomes. In the case of stunting and anaemia, it seems that the consumption of any ASF reduces the risks more than the consumption of two or three different types of ASF. These results contrast with Krasevec et al.’s (2017) finding that the consumption of more ASF types reduced stunting rates more than of just one type, whether because it was indicative of higher ASF intake or because different ASF micronutrient profiles were conducive to growth through different pathways. Nevertheless, the odds of children being underweight do appear to be lowered the most in the case of children fed both flesh and dairy foods, suggesting that in the case of underweight the rationale proposed by Krasevec et al. (2017) may indeed hold in this sample.

**Fig. 3:**
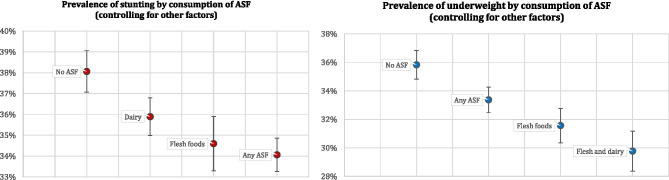
**a**, **b** Different animal-sourced foods and children’s nutrition outcomes. Figures display effects estimated from regressions in Table 3

Figure 3 displays the links between the consumption of ASF and children’s nutrition outcomes visually. Figure 3a, focused on stunting, demonstrates that while 38% children fed no ASF in the day prior to the survey were stunted, feeding children dairy products, while controlling for other factors, reduced that percentage to 36, flesh foods to 35, and any ASF to 34—an 11% reduction in the odds ratio. Figure 3b, focused on underweight, shows that 36% children fed no ASF in the 24 h prior to the survey were underweight. In contrast, *ceteris paribus*, 33% children fed any ASF, 32% children fed flesh foods, and ‘only’ 30% children fed both dairy and flesh foods were underweight—a 17% reduction in the odds ratio.

### Other determinants of children’s nutrition status

Before examining in more detail some drivers of the feeding habits linked with better nutrition outcomes, I look briefly at other factors that influence children’s nutrition status in addition to IYCF (Table 2). From child-level control variables, gender, age, and birth order are all significantly associated with nutrition outcomes. Girl children, despite being traditionally on average comparatively neglected by Indian parents (e.g. Diamond-Smith et al., 2008; Fledderjohann et al., 2014; George et al., 2000; Lone et al., 2019), are less likely to be malnourished than male children between the ages of 6 months and 2 years. This fits with previous research findings from India as well as other lower-income countries (Gupta et al., 2015; Jeyakumar et al., 2019; Kumar et al., 2006) and is generally explained through a higher incidence of childhood morbidity in male children (e.g. Haile et al., 2016, p. 11). However, beliefs around the importance of supplementary toddler feeding likely play a role here as well, as the next section suggests. The effect of age is more heterogeneous, with the prevalence of stunting and underweight higher in the older age groups but of wasting lower. Children born prematurely and higher-order children, particularly if their interval with the preceding sibling is small, are more likely to be malnourished. These results all align with existing literature, unlike findings on pre-lacteal feeds and not being breastfed within one hour of birth, which appear mostly insignificant here.^11^

Turning to household and communal-level factors, children with older, more educated mothers, in bigger and wealthier households, tend to be better nourished. Regarding caste and religion, upper-caste children and children from ‘other-religion’ families have the lowest odds of being malnourished, even after controlling for wealth. Households surveyed in the summer and monsoon seasons are more likely to have malnourished children than those surveyed in the autumn and winter.^12^ Finally, children in households with access to private improved toilets are less likely to be stunted, underweight, wasted as well as anaemic than children in households without such access. The toilet prevalence at the district level matters too—districts with higher proportion of private improved toilets have statistically lower rates of malnourished children. The findings on the links between sanitation and children’s nutrition align with recent research highlighting the crucial importance of improved toilets by e.g. Spears et al. (2013) or Hammer and Spears (2016), and underline the importance of public sanitation programmes such as India’s recently concluded Swachh Bharat that aimed to eradicate open defecation. From the other communal factors examined, children in urban areas appear at lower risk of being anaemic while children in coastal districts at lower risk of being stunted.

## Mechanisms: what encourages better IYCF practices?

Based on the analysis thus far, the IYCF factors with the most positive links with children’s nutrition are weaning at 6 months of age and feeding children semisolid complementary food of sufficient dietary diversity, ASF, and vitamin-A-rich fruits and vegetables. Before investigating whether livestock ownership and ICDS participation have any bearing on these positive child-feeding practices and in turn children’s nutrition outcomes, I first briefly look at how IYCF varies by some key demographic, socio-economic, and communal-level factors.

### Feeding practices across different factors

Table 4 displays the proportions of children following the IYCF practices most closely linked with better nutrition outcomes (weaned, minimum dietary diversity, and consumption of animal-sourced and vitamin-A-rich food) across different child characteristics (gender, age, and birth order), maternal and household characteristics (maternal education level and household caste, religion, and wealth index quintile), and communal characteristics (urban versus rural, coastal versus inland). As an additional communal characteristic, the country’s regions are added, with Indian states divided into Southern, North-Eastern, Eastern, Northern, Central, and Western regions.^13^ The link between timing of the survey and IYCF is also explored—the season of the survey because in India, most ASF is traditionally believed to be more suitable for consumption in colder seasons (e.g. Sabharwal, 2014), and day of the week when the survey was taken, because in some Hinduist traditions, non-vegetarian foods (flesh and eggs) are not eaten on Tuesdays, Thursdays, and Saturdays (Khara et al., 2020).

**Table 4:** Proportions of IYCF practices across different factors

IYCF practices identified as beneficial	Weaned (fed semisolid food) (%)	Satisfactory diet. Diversity (%)	Any animal-sourced foods (%)	Vit.A-rich fruits and vegetables (%)	*N*
Gender					
Female	**76.8**	**20.4**	**35.8**	**40.6**	*27,248*
Male	76.5	20.3	35.6	39.9	*29,773*
Age					
6–11 months	57.1	10.2	26.0	21.4	*19,763*
12–17 months	84.1	23.0	38.6	45.7	*19,252*
18–23 months	**90.0**	**28.7**	**43.1**	**54.8**	*18,006*
Birth order					
First or second	**77.5**	**22.0**	**38.8**	**40.8**	*38,511*
Third of more	74.7	16.7	28.5	38.9	*18,510*
Mother’s education					
No education	71.9	14.1	24.6	36.6	*16,283*
Incomplete primary	78.4	18.7	35.1	40.9	*3425*
Complete primary	76.0	16.7	30.7	38.3	*4533*
Incomplete secondary	77.6	22.5	38.7	41.3	*21,688*
Complete secondary	80.0	25.9	44.8	42.8	*5225*
Higher	**81.9**	**27.7**	**49.7**	**44.4**	*5867*
Household caste					
SC	75.9	20.5	35.0	39.3	*11,300*
ST	**77.7**	19.8	31.0	**44.6**	*11,963*
OBC	76.7	20.1	35.6	39.7	*23,241*
UC	77.0	**21.3**	**39.4**	40.2	*10,134*
Household religion					
Hindu	76.7	19.8	34.7	40.4	*42,271*
Muslim	74.9	21.3	37.1	37.8	*7615*
Other	**80.5**	**26.9**	**47.9**	**44.0**	*7135*
Household wealth index quintile					
Lowest	72.4	15.2	23.7	38.9	*14,552*
Lower middle	75.4	18.5	31.1	39.6	*13,190*
Middle	77.6	21.2	38.5	39.5	*11,620*
Upper middle	79.4	**25.2**	44.5	**42.2**	*9667*
Highest	**80.9**	24.9	**48.2**	41.8	*7982*
Season of interview					
Winter	75.3	19.0	32.0	41.1	*7561*
Summer	76.2	21.2	**37.4**	40.2	*29,921*
Monsoon	77.8	19.1	33.8	39.8	*18,351*
Autumn	**81.1**	**23.6**	36.8	**43.5**	*1188*
Day of interview					
Monday	75.8	19.3	35.4	39.9	*8183*
Tuesday	76.1	19.7	34.6	39.3	*8005*
Wednesday	**77.5**	20.6	**37.1**	40.5	*8227*
Thursday	75.7	19.8	34.4	39.5	*8254*
Friday	77.4	21.2	36.4	**41.5**	*8314*
Saturday	76.6	**21.7**	36.9	40.9	*8186*
Sunday	77.3	20.2	35.0	39.8	*7852*
Area					
Urban	**79.8**	**25.3**	**44.7**	**42.4**	*13,492*
Rural	75.5	18.6	32.4	39.4	*43,529*
Coastal	**82.5**	**32.6**	**53.1**	**47.0**	*3800*
Inland	75.8	18.6	33.2	39.2	*53,221*
Region					
Southern	**83.5**	**37.9**	**61.9**	44.6	*5544*
North-Eastern	82.5	32.6	49.4	**50.1**	*8007*
Eastern	75.6	22.2	36.4	44.8	*12,572*
Northern	72.1	13.7	34.7	32.9	*9080*
Central	74.3	10.8	20.7	35.8	*17,876*
Western	77.7	17.4	29.5	39.4	*3942*

Bold numbers indicate the highest proportion for that outcome variable within each factor examined

The cross-tabulations show similar patterns to the findings vis-à-vis the effect of control variables on children’s nutrition outcomes (Sect. 3.3). Girl children are fed slightly better than boy children. This may seem counter-intuitive given the above-mentioned average preference for boy children in India (e.g. Diamond-Smith et al., 2008; Lone et al., 2019) but is likely related to traditional beliefs that breast milk rather than food ensures good growth among toddlers (Sabharwal, 2014). Boy children in this study’s sample are indeed breastfed at statistically significantly higher rates than girl children.^14^ The belief of some mothers in the lower importance of complementary semisolid food in young children may then also in part account for the higher adherence to better feeding practices among older children in the sample. However, this discrepancy may be related to the lesser need for parents to feed older toddlers semisolid food as well, as older children are better able to feed themselves. That could partially explain why further-order children are fed less well than the first or second-born ones—their parents may have less time to devote to their feeding. In addition, larger families are likely to be financially poorer in per-capita terms than smaller families.

From the other characteristics analysed, more children of mothers with higher education and living in richer households follow the beneficial feeding habits than other children, which aligns with lower malnutrition rates in those groups. The same is true of children from ‘other’ religions (Christianity, Sikhism, Buddhism etc.) and from upper-caste backgrounds. The positive feeding habits seem to be followed by children most in the autumn, which again lines up with the finding of positive associations between the surveys taken in the autumn and winter and better children’s nutrition outcomes. However, the feeding practices show no clear pattern based on the day of week when the surveys were conducted. Finally, children in urban and coastal areas and in the Southern and North-Eastern regions are fed proportionally better than children in rural and inland areas and children in the Central and Northern regions.

### Effects of livestock ownership

Turning to the examination of whether livestock ownership and participation in the ICDS encourage the IYCF habits linked with better nutrition outcomes, based on both theory and existing research one could expect owning animals to be positively associated with greater consumption of different ASFs. However, since livestock ownership may also lead to higher occurrence of enteric diseases (e.g. Kaur et al., 2017), the greater ASF consumption might not translate into improved nutrition outcomes. I examined these relationships by regressing the household and district-level measures of cattle and poultry ownership on the consumption of any ASF, flesh foods, dairy products (cattle), and eggs (poultry) as well as on the four key nutrition outcomes—being stunted, underweight, wasted, and anaemic.^15^

The results, displayed in Table 5, suggest that the ownership of cattle either at the household or district level is not conducive to better child-feeding practices or nutrition outcomes. Household cattle ownership has mostly insignificant links with the outcome variables analysed. Even the consumption of dairy products is not significantly higher among cattle-owning households than among non-cattle-owning ones. This contrasts with previous research findings (e.g. Choudhury & Headey, 2018) and could be due to the lack of disaggregation between cattle owned for ploughing and other farm work and dairy cattle.^16^ District-level prevalence of cattle ownership is meanwhile associated with significantly lower consumption of ASF and higher rates of child malnourishment, even when controlling for the host of other determinants included.

**Table 5:** Livestock ownership and children’s feeding habits and nutrition outcomes

Livestock ownership	Any ASF	Flesh foods	Eggs (poultry), dairy (cows)	Stunted	Underweight	Wasted	Anaemic dairy (cows)
Cattle—individual	0.974	0.931*°*	1.038	1.030	1.038	1.078*	1.010
	0.022	0.034	0.025	0.030	0.032	0.035	0.028
Cattle—district	0.223***	0.060***	0.436***	1.171*°*	1.491***	1.632***	1.611***
	0.037	0.015	0.071	0.167	0.164	0.194	0.201
Poultry—individual	1.174***	1.180***	1.261***	0.986	0.988	1.041	0.988
	0.034	0.044	0.045	0.022	0.023	0.026	0.020
Poultry—district	4.556***	5.332***	1.841***	0.664***	0.488***	0.575***	0.657***
	0.629	1.316	0.196	0.055	0.047	0.059	0.069

The numbers next to the variables are AORs, below are standard errors. The N for all regressions is 57,021

The models were estimated using multilevel logistic regressions with the first level individual/household, second level the district, and third level the Indian state. Where the dependent variables are nutrition outcomes, the regressions were the same as those in Table 2 but with the addition of the livestock variables. The models examining links between livestock ownership and the consumption of different foods were estimated similarly but with the omission of most IYCF variables except for breastfeeding as control variables

***p < 0.001; **p < 0.01; *p < .05; °*p* < 0.1

**Fig. 4:**
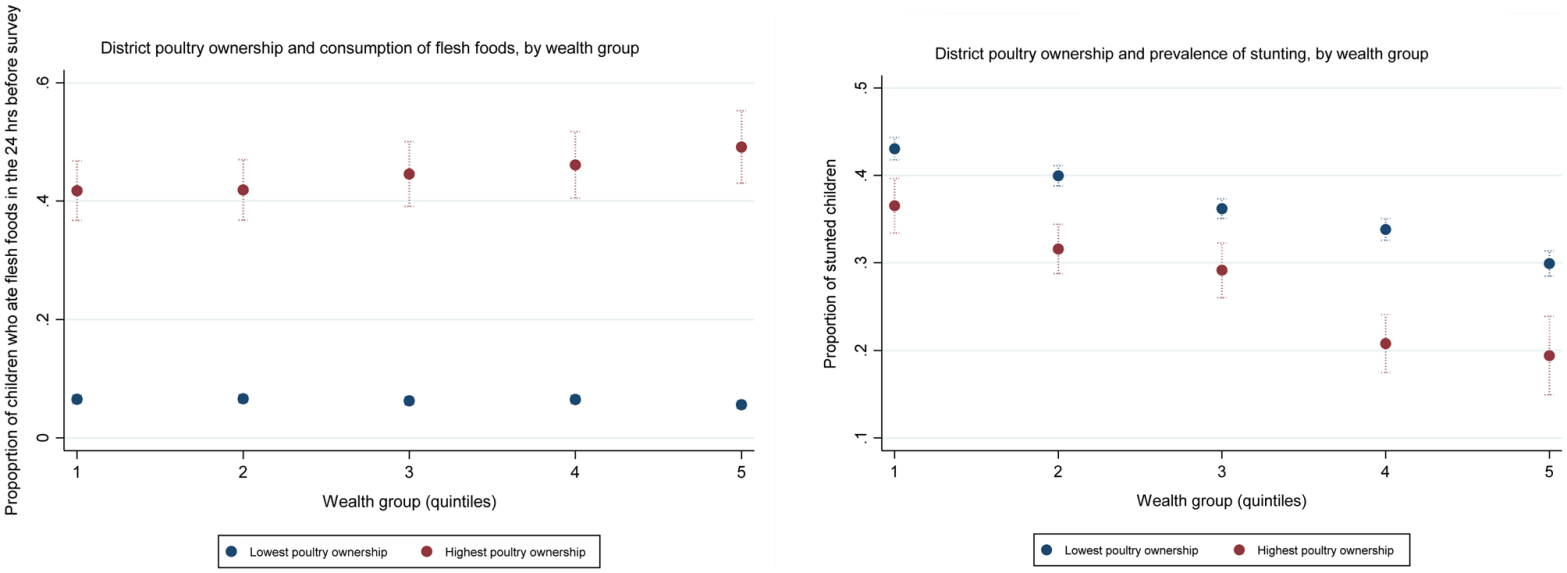
**a**, **b** District-level poultry ownership and child consumption of flesh foods and stunting rates. Marginal effects from regressions akin to those in Table 5, with the addition of an interaction term between district-level poultry ownership and households’ wealth-index quintile

Unlike cattle ownership, the ownership of poultry, at the household but particularly at the district level, seems to significantly increase the odds of positive feeding habits and lower the likelihood of child malnutrition. Household poultry ownership is associated with a higher probability of children being fed any ASF, flesh foods as well as eggs albeit not with a significantly lower risk of malnourishment. The district-level prevalence of poultry ownership has in contrast very strong links with both a higher consumption of ASF and reduced rates of all four types of child malnutrition. This is true across different wealth groups—Fig. 4 shows that in all wealth quintiles, going from minimal district-level poultry ownership (0%) to a maximum one (97%), while holding other factors constant, is associated with a four-to-six-time increase in children’s rate of consuming flesh foods and a 10-to-40-percentage reduction in their stunting prevalence.^17^

### Effects of ICDS

The other key mechanism that could contribute to Indian children’s better feeding habits and thus also better nutrition outcomes is participation in the ICDS, a national programme expressly aimed at supporting good feeding, hygienic, and health practices among pre-school-aged children. The specific ICDS services examined in this study include the receipt of food from the programme (daily and at least weekly) and full programme participation (food at least weekly, health check-ups and weigh-ins monthly, and regular pre-school attendance). The food receipts, if varied in their composition, could in theory easily contribute to better dietary diversity and higher consumption of ASF and vitamin-A-rich fruits and vegetables while full programme participation was found by Gragnolati et al. (2006) to be most beneficial to children’s nutrition status. The effects of the programme’s services on children’s IYCF (weaned, satisfactory dietary diversity, and consumption of any ASF and of vitamin-A-rich fruits and vegetables) and nutrition outcomes (stunted, underweight, wasted, and anaemic) were estimated using PSM—the nearest-neighbour and five-nearest-neighbours’ approaches—and the results are shown in Table 6.^18^

**Table 6:** ICDS participation and children’s feeding habits and nutrition outcomes

ICDS	N off support	N treated (on support) N untreated (on support)	Weaned (fed semisolid food)	Satisf. diet. diversity	Any ASF	Vit.A-rich fruits and vegetables	Stunted	Underweight	Wasted	Anaemic
Food daily										
Nearest neighbour	*59*	*9340*	− 0.023**	0.019˚	0.027**	0.013	0.001	− 0.022˚	0.009	0.000
		*47412*	0.010	0.010	0.012	0.011	0.012	0.011	0.010	0.012
Five nearest neighbours	*59*	*9340*	− 0.010	0.019**	0.025**	0.016˚	0.008	− 0.007	0.008	0.008
		*47412*	0.008	0.008	0.009	0.010	0.009	0.009	0.008	0.010
Food at least weekly										
Nearest neighbour	*109*	*18301*	− 0.009	0.002	0.008	0.011	0.017	0.004	− 0.008	− 0.007
		*38401*	0.009	0.009	0.010	0.011	0.011	0.010	0.009	0.011
Five nearest neighbours	*109*	*18301*	− 0.009	0.013˚	0.013	0.013	0.026**	0.003	− 0.007	− 0.009
		*38401*	0.008	0.007	0.009	0.009	0.009	0.009	0.008	0.009
Full participation										
Nearest neighbour	*0*	*38401*	− 0.002	0.050***	0.050***	0.060***	0.011	0.016	0.017	0.001
		*52844*	0.010	0.010	0.012	0.012	0.012	0.011	0.011	0.012
Five nearest neighbours	*0*	*3967*	− 0.002	0.050***	0.050***	0.060***	0.016˚	0.013	0.016	0.002
		*52844*	0.008	0.008	0.009	0.010	0.009	0.009	0.010	0.009

The numbers next to the variables are the Average Treatment on the Treated (ATT), below are standard errors

The models were estimated using propensity score matching (PSM) with the nearest-neighbour and five-nearest-neighbours’ approaches

***p < 0.001; **p < 0.01; *p < .05; °*p* < 0.1

They show that daily receipts of food and full participation in the programme are both statistically significantly associated with improved IYCF, particularly minimum dietary diversity and consumption of ASF. Full ICDS participation raises the likelihood of children eating vitamin-A-rich fruits and vegetables as well. Nevertheless, only in the case of daily food receipts there is an indication that the better feeding habits have translated also into improved nutrition outcomes, with ICDS beneficiaries slightly less underweight than non-beneficiaries.

**Fig. 5:**
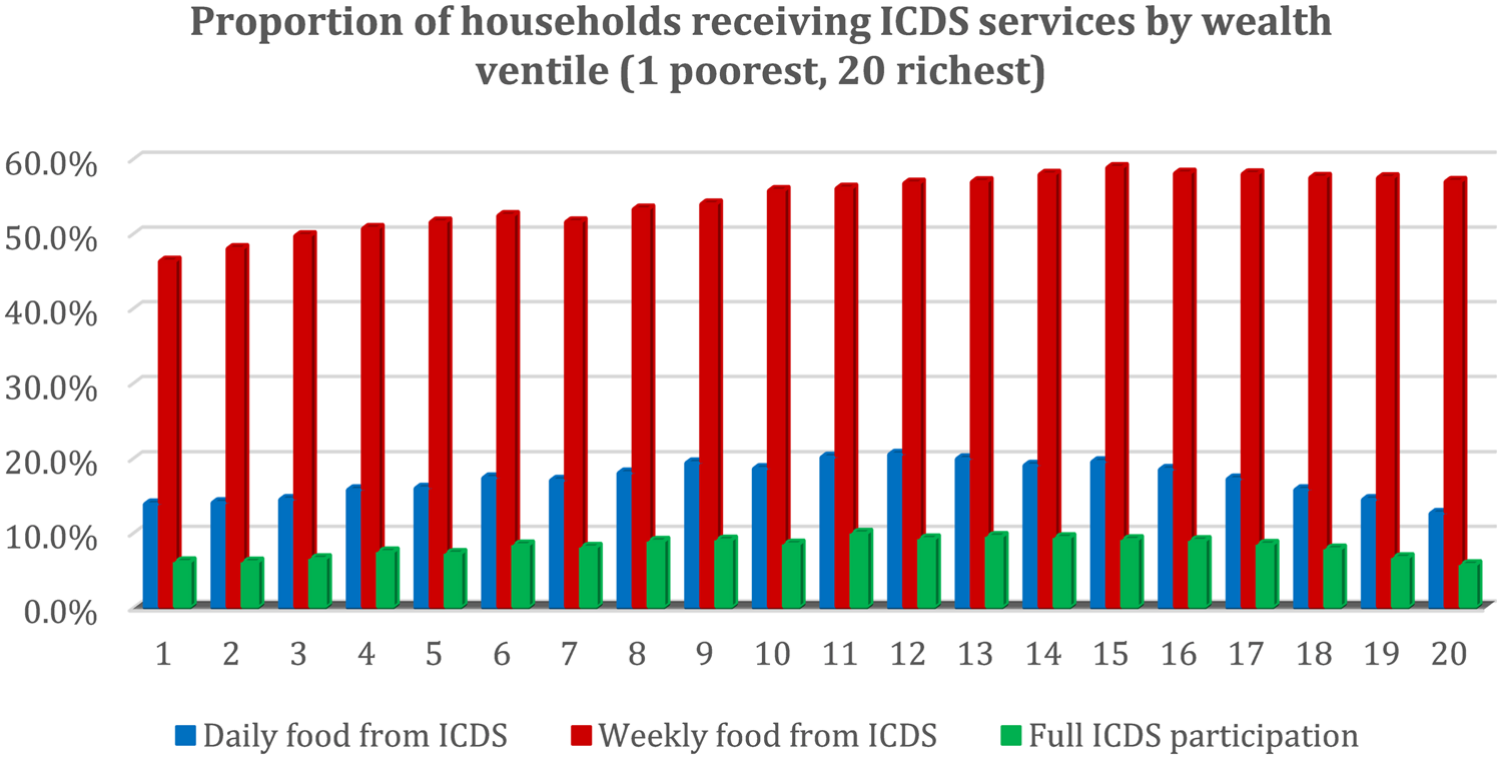
Receipts of ICDS services by wealth ventile. Proportions estimated by calculating the percentage of households in each wealth-index ventile (20th) receiving each type of ICDS service examined

One underlying reason may be the programme’s relatively regressive targeting, with middle-wealth households more likely to participate in all three services analysed than the lowest-wealth households (Fig. 5). The low coverage of the poorest households is, according to Chakrabarti et al. (2019), primarily due to a worse performance of the programme in India’s poorer districts rather than due to discrimination against lower-income or lower-caste households in specific areas. Improving the programme’s performance and reach in India’s poorer states and districts would thus enable the ICDS to attain more sizeable positive nutrition effects, particularly since children in such districts tend to suffer from malnourishment proportionally the most.

## Discussion and conclusions

Despite India’s rapid economic growth over the past several decades, the country’s child malnutrition rates have remained higher than those of many economically poorer countries. Moreover, partial results from the most recent round of the National Family Health Survey (NFHS V 2019–2020) indicate that by 2019, the gradual albeit relatively slow progress in improving nutrition outcomes among Indian children largely stalled. The Covid-19 pandemic, which has led to an economic decline along with a reduction in the provision of many child-focused programmes in India, is widely expected to contribute to further deterioration on this front (Headey et al., 2020; Khullar & Sharma, 2020). Consequently, it is of key importance to continue deepening our understanding of the different drivers of Indian children’s nutrition outcomes. This study analysed nationally representative data from NFHS IV (2015–2016) on more than 57,000 Indian toddlers to assess the links between children’s feeding practices and malnutrition rates. The study’s key contribution to existing literature lies in its detailed examination of the effects of different types of weaning food, with a particular focus on ASF, and its interrogation whether livestock ownership and ICDS participation may nurture beneficial feeding habits and positive nutrition outcomes.

The study’s baseline models investigated the connection between four WHO-recommended IYCF practices—weaning/complementary semisolid feeding, minimum dietary diversity, minimum dietary frequency, and continued breastfeeding—and children’s rates of stunting, underweight, wasting, and anaemia. The key conclusion, largely in line with existing research, was that feeding children older than 6 months semisolid food of sufficient dietary diversity was strongly associated with better nutrition outcomes. Starting the complementary feeding prior to 6 months of age did not seem harmful as suggested by some previous research (Bhutia, 2014; Gupta et al., 2015) but nor did it appear beneficial as proposed by others (Teshome et al., 2009; Vyas et al., 2014). Socio-economic variables were found to play a mediating role in the observation of recommended IYCF, with wealthier households and more educated mothers more likely to wean their children on time and feed them more diverse food. Nevertheless, much of the inappropriate practice in child feeding cut across socio-economic lines, with 75% households even in the wealthiest quintile and 72% mothers with higher education not providing their toddlers with the WHO-recommended minimum diverse diet (Table 4).

The study’s central inquiry was into the links between different types of semisolid food consumed and young children’s nutrition outcomes. The clear limitation in this regard was that the pertinent data were limited to information about the children’s diets in the preceding day and only referred to the types of food consumed, not to their quantity. Nevertheless, the sample of children examined was very large, which should to some extent alleviate concerns that the children’s reported diets from the previous 24 h may not represent broader dietary trends. The key discoveries in this part of the study included significantly lower odds of being underweight and wasted among children who had consumed vitamin-A-rich fruits and vegetables and significantly lower likelihood of being stunted, underweight, and anaemic among children who had consumed any ASF. The findings on the importance of ASF were particularly strong and aligned with results from studies conducted in other-country contexts (e.g. Choudhury & Headey, 2018; Headey et al., 2018, Krasevec et al., 2016), which indicated that ASF’s high protein and micronutrient (vitamin D, calcium, and zinc) contents made it an important contributor to reduced child malnutrition rates. Previous research on the relationship between ASF and children’s nutrition outcomes also suggested that the consumption of several types may be even more positive, either because it is indicative of a larger quantity of ASF consumed or because ASFs’ varied nutrient profiles may boost nutrition through diverse pathways (Krasevec et al., 2017). This study did not validate this hypothesis in most of the nutrition outcomes examined but did find that the odds of being underweight were reduced the most for children who had consumed both flesh and dairy foods the day before the survey (17% reduction in the odds of being underweight, compared to 6% reduction for only dairy and 12% reduction for only flesh foods’ consumption).

The remainder of the study concentrated on closer examination of factors that could support the feeding habits identified as linked with children’s better nutrition outcomes—i.e. timely weaning, minimum dietary diversity, and the consumption of vitamin-A-rich fruits and vegetables and ASF. Correlations between IYCF and key indicators (Table 4) revealed that most factors aligned with better children’s nutrition outcomes also aligned with closer observation of beneficial feeding practices. First and second-born children of more educated mothers, from wealthier, upper-caste households, in urban areas, particularly in the Southern and North-Eastern regions of India were fed more appropriately than their counterparts. However, and on the surface perhaps surprisingly, while a significantly higher proportion of male than female children in the sample were still breastfed, female children received better complementary feeds.

Further analysis focused in greater detail on the role of two potential ‘remedies’—participation in the ICDS national programme, targeted specifically at improving children’s nutrition and health, and household and communal-level livestock ownership. Children who ‘fully’ participated in the ICDS (received food rations, medical check-ups and weigh-ins, and attended preschool) and who received daily food rations from the programme were found to have significantly higher odds of consuming supplementary food of minimum dietary diversity as well as animal-sourced and vitamin-A-rich food. Nevertheless, only in the case of the daily food rations this appears to have translated into lower malnutrition rates (i.e. reduced odds of being underweight). Meanwhile, whereas the ownership of cattle neither on the household nor on the district level had any positive association with better IYCF or nutrition status, poultry ownership did. Household-level ownership of poultry was linked with a significantly higher consumption of meat, eggs, and ASF in general. However, it was particularly district-level ownership of poultry that turned out to have a very strong association with all the positive IYCF and nutrition outcomes examined—for example, districts with the highest level of poultry ownership (97%) compared to the lowest level (0%) observed had, *ceteris paribus*, five-times higher consumption of flesh foods among children, 32% lower prevalence of child stunting, and 51% lower prevalence of child underweight (Fig. 4).

These results have several implications for policy and research. First, even accounting for the potential influence of confounding unobservable characteristics in the relationship between IYCF and children’s nutrition status, it seems probable that if more Indian children followed the IYCF practices identified as beneficial, their malnutrition rates would decline. Naturally, socio-economic factors play a significant role in whether a household can afford to feed children appropriately as the higher prevalence of good feeding practices among wealthier households clearly demonstrated.^19^ Nevertheless, the need for a greater awareness of positive child-feeding habits in India has also become apparent. That the majority of even the wealthiest households in the study did not abide by the recommended IYCF and that even though traditionally boy children are preferred to girl children in India (e.g. Diamond-Smith et al., 2008; Lone et al., 2019), girl children were found to have been fed significantly better than male children—who were on the other hand breastfed longer—attest to that. This aligns with existent research suggesting that many Indian caregivers still hold some traditional erroneous beliefs about child feeding. These consider breast milk the most nutritious food for children under 2 years of age, with complementary semisolid food only of secondary importance (Sabharwal, 2014). In fact, some caregivers deem complementary feeding altogether unnecessary prior to children reaching 1 year of age and afterwards, a few spoonfuls of rice or dal water in addition to breast milk may be seen as sufficient (Vyas et al., 2014). Meat, eggs, pulses, and even vegetables are often depicted as unsuitable weaning foods on which children could choke (Manikam et al., 2018; Sabharwal, 2014).

The awareness and practice of the child-feeding practices associated with better nutrition outcomes varies along individual factors—e.g. mothers with higher education feed their children better than less educated mothers—but the study results have also indicated that the feeding beliefs and practices exhibit a spatial pattern (as suggested also by e.g. Patel et al. 2012). For example, ASF was found to be fed to toddlers at five-times-higher rates in districts with the highest prevalence of poultry ownership than in districts with the lowest prevalence. Part of the underlying explanation might be that in areas where a lot of poultry is raised, ASF (particularly meat and eggs) is cheaper and/or more accessible. However, a greater consumption of dairy products was not correspondingly observed in areas with higher prevalence of cattle ownership. This highlights the likelihood that it is greater consideration of meat and eggs as appropriate food for young children in areas with higher poultry ownership that underlines their children’s seemingly better feeding habits and nutrition outcomes even more than the potentially lower costs of such food.^20^ Such normalisation of ASF as well as of fruits and vegetables as suitable for complementary feeding of toddlers should be encouraged throughout all of India. The ICDS, with its existing vast network of *anganwadi* centres, may be currently in the best position to provide it.^21^ The results vis-à-vis the ICDS services and children’s feeding habits are already somewhat encouraging in this regard, as they show that children participating in the programme are more likely to be fed a minimum diverse diet, ASF as well as vitamin-A-rich fruits and vegetables—likely due to a combination of the food rations and parental nutrition education received from the programme. Ensuring that the ICDS covers greater proportions of the lowest-income households with its most comprehensive package of services and that the nutrition education imparted focuses on wider promotion of feeding habits identified as beneficial here could be instrumental in this endeavour (as also argued by Chakrabarti et al., 2019). It would also undoubtedly require increasing the amount of funding that the programme receives from the national government along with the wages of its employees, with a particular attention paid to poorer Indian districts in Central and Northern parts of the country (Chakrabarti et al., 2019; Singh, 2019).

The final implication of the study’s results, more research than policy-related, concerns the relationship between IYCF and nutrition outcomes. The study demonstrated that the links between the two elements are clearly strong but also complex. While household poultry ownership and some forms of ICDS participation in the study were robustly associated with beneficial feeding habits, their odds of improving children’s nutrition outcomes were largely insignificant. In the case of household poultry ownership, the absence of a more robust connection with good nutrition outcomes despite the children’s better feeding habits could be partially explained through the frequently greater occurrence of enteric diseases in poultry-keeping households due to the higher likelihood of children ingesting poultry faeces (Kaur et al., 2017; Ngure et al., 2014). Children in such households may thus be more likely to receive more nutritious diets but be unable to fully utilise all the nutrients due to greater morbidity from diarrhoea, environmental enteropathy, and other diseases.^22^ However, the lack of a stronger relationship could also have to do with this study’s inability to control for the quantities of different foods consumed. Similarly, when it comes to the ICDS, the reason for the failure to discover a stronger connection between participation and lower malnutrition rates despite improved feeding practices could have arisen due to the inability of the PSM to control for unobservable characteristics (e.g. Kandpal, 2011) or due to the nature of the NFHS IV data where controlling for the length of time of ICDS participation and the quantities of different food types consumed is impossible. More specific, longitudinal research on these topics could thus help shed more insight into the links between IYCF practices and children’s nutrition outcomes in India and beyond and by extension help engender more nuanced recommendations.

This article aimed to deepen existing understanding of the relationship between India’s internationally high child malnutrition rates and IYCF practices, with a particular focus on the types of semisolid complementary food provided. The findings highlighted the importance of diverse complementary feeds, including particularly animal-sourced and vitamin-A-rich foods, in reducing the risks of malnourishment among 6-to-23-month-old children, and the positive links between the observation of such feeding practices on the one hand and poultry ownership and ICDS food rations on the other. The key policy recommendation that emerged from the study relates to the importance of greater caregivers’ awareness of and adherence to useful child-feeding practices, which could potentially be facilitated by strengthening the ICDS’ nutrition components. However, longitudinal studies gathering detailed data on the types and quantities of different complementary foods consumed by children and on their nutrition status could help yield further, more specific results.

### Electronic supplementary material

Below is the link to the electronic supplementary material.

Supplementary file1 (DOCX 313 kb)

## Data Availability

Data are publicly available.

## Appendix 1

See Tables 7, 8, and 9.

**Table 7:** Sensitivity test 1: main empirical models from Table 2 estimated with logistic regressions with district-clustered standard errors

Nutrition outcome	Stunted		Underweight		Wasted		Anaemic	
Model	Full	No soc.-econ	Full	No soc.-econ	Full	No soc.-econ	Full	No soc.-econ
Infant & young child feeding	0.954˚	0.947*	0.899***	0.884***	0.877***	0.875***	0.996	0.993
Weaned (fed semisolid food)	0.025	0.025	0.025	0.024	0.025	0.025	0.023	0.023
	0.915***	0.937**	0.940*	0.909***	0.992	0.977	0.977	0.967
Minimum dietary diversity	0.023	0.023	0.025	0.024	0.030	0.029	0.025	0.024
	0.985	0.987	0.989	0.978	1.020	1.010	1.015	1.015
Minimum dietary frequency	0.022	0.022	0.023	0.022	0.026	0.025	0.021	0.020
Breastfed	1.045	1.102**	1.222***	1.269***	1.136***	1.162***	0.937*	0.951˚
	0.032	0.033	0.040	0.041	0.041	0.041	0.025	0.025
Child characteristics								
Female child	0.775***	0.784***	0.786***	0.795***	0.881***	0.882***	0.985	0.990
	0.014	0.014	0.015	0.015	0.019	0.019	0.016	0.016
Age (comp. to 6–11 months)								
12–17 months	2.118***	2.116***	1.295***	1.309***	0.894***	0.891***	0.998	1.001
	0.051	0.049	0.033	0.032	0.024	0.024	0.020	0.020
18–23 months	3.100***	3.112***	1.699***	1.712***	0.771***	0.769***	1.011	1.017
	0.087	0.086	0.046	0.045	0.023	0.022	0.023	0.023
Preterm	1.202***	1.188***	1.190***	1.196***	1.096*	1.096*	1.028	1.021
	0.048	0.046	0.049	0.048	0.047	0.046	0.039	0.038
Any prelacteal feed	1.006	0.957˚	0.969	0.944*	0.991	0.978	1.017	1.010
	0.025	0.023	0.028	0.027	0.031	0.030	0.025	0.025
Breastfed within 1 h of birth	1.031	1.049*	1.029	1.033	1.020	1.023	0.966	0.974
	0.023	0.023	0.025	0.024	0.026	0.026	0.021	0.022
Had diarrhoea in the last 2 weeks	1.006	0.992	1.127***	1.131***	1.110***	1.114***	1.036	1.035
	0.027	0.026	0.032	0.031	0.033	0.033	0.026	0.026
Birth order	1.077***	1.154***	1.070***	1.158***	1.033**	1.068***	1.0121.039***	
	0.011	0.011	0.011	0.012	0.011	0.011	0.009	0.009
Birth interval	0.998***	0.998***	0.997***	0.997***	0.998***	0.998***	1.000	1.000
	0.000	0.000	0.001	0.000	0.000	0.000	0.000	0.000
Maternal characteristics								
Mother’s age at birth	0.986***	0.977***	0.987***	0.978***	1.003	0.999	0.996˚	0.993**
	0.003	0.003	0.003	0.003	0.003	0.003	0.003	0.003
Mother undernourished	1.381***	1.470***	1.886***	2.018***	1.493***	1.543***	1.198***	1.234***
	0.032	0.033	0.043	0.045	0.035	0.035	0.028	0.029
Mother’s education in years	0.964***		0.965***		0.989***		0.986***	
	0.002		0.003		0.003		0.002	
Household characteristics								
HH size	0.988**	0.973***	0.986***	0.972***	0.992˚	0.986***	1.009*	1.003
	0.004	0.004	0.004	0.004	0.004	0.004	0.004	0.004
Female head of HH	1.018	1.003	0.995	1.000	0.957	0.962	0.983	0.976
	0.029	0.028	0.029	0.029	0.031	0.031	0.026	0.026
Number of children under 5 in HH	1.089***	1.106***	1.066***	1.085***	1.012	1.018	1.006	1.011
	0.016	0.016	0.015	0.015	0.016	0.015	0.012	0.012
Caste (comp. to scheduled caste)								
Scheduled tribe	0.847***		1.077˚		1.203***		1.323***	
	0.030		0.043		0.050		0.054	
Other backward caste	0.883***		0.896***		0.982		0.919**	
	0.025		0.025		0.028		0.024	
Upper caste	0.736***		0.785***		0.942		0.877***	
	0.026		0.030		0.038		0.028	
Religion (comp. to Hindu)								
Muslim	1.049		1.002		0.975		0.900**	
	0.037		0.036		0.041		0.030	
Other (Sikh, Christian, Buddhist, Traditional…	0.835***		0.936		0.926		0.863**	
	0.040		0.054		0.061		0.049	
Wealth quintile (comp. to lowest)								
Lower middle	0.897***		0.876***		0.909**		0.981	
	0.025		0.024		0.028		0.027	
Middle	0.838***		0.752***		0.838***		0.992	
	0.028		0.025		0.030		0.032	
Upper middle	0.731***		0.659***		0.781***		0.952	
	0.025		0.025		0.031		0.034	
Highest	0.674***		0.536***		0.691***		0.900*	
	0.028		0.024		0.036		0.037	
Season (comp. to winter)								
Summer	1.072	1.077	1.288***	1.289***	1.265***	1.276***	1.444***	1.470***
	0.051	0.049	0.051	0.051	0.063	0.064	0.071	0.072
Monsoon	1.205***	1.209***	1.693***	1.672***	1.580***	1.565***	1.786***	1.791***
	0.060	0.061	0.073	0.072	0.084	0.083	0.100	0.099
Autumn	1.178*	1.228*	1.443***	1.440***	1.304*	1.365*	1.617***	1.576***
	0.089	0.104	0.139	0.127	0.172	0.194	0.193	0.158
Private improved toilet	0.904***	0.728***	0.891***	0.681***	0.928**	0.804***	0.913***	0.829***
	0.021	0.016	0.021	0.015	0.024	0.018	0.020	0.017
Communal characteristics								
District toilet prevalence	0.817**	0.596***	0.645***	0.446***	0.767*	0.638***	0.787*	0.668***
	0.060	0.068	0.068	0.046	0.097	0.078	0.095	0.079
Urban	0.952˚		1.033		1.023		0.978	
	0.027		0.027		0.033		0.024	
Coastal	0.891˚		0.827**		1.050		1.040	
	0.061		0.058		0.063		0.066	

All regressions were estimated using standard logistic regressions controlling for the Indian state of residence, with robust standard errors clustered by districts. The numbers next to the variables are adjusted odd ratios (AOR), below are standard errors. The N for all regressions is 57,021

****p* < 0.001; ***p* < 0.01; **p* < .05; °*p* < 0.1

**Table 8:** Sensitivity test 2: Main empirical models from Table 2 with p values and False Discovery Rate-sharpened q values

Part 1								
Nutrition outcome	Stunted		Underweight		Wasted		Anaemic	
Model	Full	No soc.-econ	Full	No soc.-econ	Full	No soc.-econ	Full	No soc.-econ
Infant & young child feeding								
Weaned (fed semisolid food)	0.919	0.900	0.892	0.876	0.874	0.874	0.993	0.990
	0.028*	0.005**	0.000 ***	0.000 ***	0.000 ***	0.000 ***	0.759	0.673
	0.021*	0.004**	0.001 ***	0.001**	0.001**	0.001**	0.320	0.256
Minimum dietary diversity	0.912	0.865	0.902	0.872	0.956	0.942	0.966	0.954
	0.017*	0.000***	0.000 ***	0.000 ***	0.123	0.035*	0.143	0.035*
	0.014*	0.001**	0.001**	0.001**	0.074˚	0.023*	0.085˚	0.023*
Minimum dietary frequency	1.014	1.014	0.987	0.976	1.017	1.007	1.016	1.018
	0.684	0.678	0.558	0.276	0.507	0.762	0.433	0.366
	0.305	0.256	0.267	0.129	0.242	0.293	0.217	0.171
Breastfed	1.133	1.208	1.210	1.263	1.126	1.148	0.934	0.950
	0.007**	0.000***	0.000 ***	0.000 ***	0.001 ***	0.000 ***	0.014*	0.057˚
	0.007**	0.001**	0.001**	0.001**	0.002**	0.001**	0.012*	0.036*
Child characteristics								
Female child	0.785	0.790	0.782	0.790	0.878	0.878	0.983	0.988
	0.000 ***	0.000***	0.000 ***	0.000 ***	0.000 ***	0.000 ***	0.310	0.456
	0.001**	0.001**	0.001**	0.001**	0.001**	0.001**	0.160	0.211
Age (comp. to 6–11 months)								
12–17 months	2.374	2.374	1.316	1.330	0.901	0.897	0.999	1.001
	0.000 ***	0.000***	0.000 ***	0.000 ***	0.000 ***	0.000 ***	0.977	0.944
	0.001**	0.001**	0.001**	0.001**	0.001**	0.001**	0.374	0.364
18–23 months	3.672	3.710	1.721	1.742	0.773	0.772	1.008	1.015
	0.000 ***	0.000***	0.000 ***	0.000 ***	0.000 ***	0.000 ***	0.736	0.511
	0.001**	0.001**	0.001**	0.001**	0.001**	0.001**	0.320	0.231
Preterm	1.248	1.270	1.175	1.185	1.063	1.065	1.027	1.021
	0.000 ***	0.000***	0.000 ***	0.000 ***	0.149	0.136	0.456	0.563
	0.001**	0.001**	0.001**	0.001**	0.088˚	0.075˚	0.222	0.541
Any prelacteal feed	0.996	0.979	0.969	0.945	0.989	0.977	1.020	1.015
	0.912	0.555	0.238	0.030*	0.690	0.402	0.394	0.517
	0.358	0.241	0.128	0.021*	0.305	0.185	0.197	0.231
Breastfed within 1 h of birth	1.066	1.050	1.014	1.017	1.008	1.011	0.954	0.963
	0.054˚	0.137	0.557	0.475	0.761	0.648	0.024*	0.065˚
	0.037*	0.075˚	0.267	0.218	0.320	0.252	0.019*	0.041*
Had diarrhoea in the last 2 weeks	1.011	1.021	1.136	1.137	1.121	1.124	1.030	1.030
	0.781	0.586	0.000 ***	0.000 ***	0.000 ***	0.000 ***	0.235	0.234
	0.320	0.241	0.001**	0.001**	0.001**	0.001**	0.128	0.118
Birth order	1.063	1.161	1.071	1.154	1.036	1.067	1.021	1.046
	0.000 ***	0.000***	0.000 ***	0.000 ***	0.001 ***	0.000 ***	0.025*	0.000 ***
	0.001**	0.001**	0.001**	0.001**	0.002**	0.001**	0.019*	0.001**
Birth interval	0.997	0.998	0.997	0.997	0.998	0.998	1.000	1.000
	0.000 ***	0.000***	0.000 ***	0.000 ***	0.000 ***	0.000 ***	0.820	0.672
	0.001**	0.001**	0.001**	0.001**	0.001**	0.001**	0.328	0.256
Maternal characteristics								
Mother’s age at birth	0.993	0.982	0.985	0.976	1.000	0.997	0.993	0.990
	0.090˚	0.000***	0.000***	0.000***	0.878	0.243	0.004**	0.000***
	0.055˚	0.001	** 0.001	** 0.001	** 0.349	0.120	0.004**	0.001**
Mother undernourished	1.367	1.458	1.904	2.032	1.515	1.562	1.197	1.231
	0.000***	0.000***	0.000***	0.000***	0.000***	0.000***	0.000***	0.000***
	0.001**	0.001**	0.001**	0.001**	0.001**	0.001**	0.001**	0.001**
Mother’s education in years	0.959		0.963		0.986		0.986	
		0.000***		0.000***		0.000***		0.000***
		0.000**		0.001**		0.001**		0.001**

Part 2								
Household characteristics								
HH size	0.989	0.979	0.988	0.975	0.994	0.988	1.010	1.003
	0.061˚	0.000 ***	0.003**	0.000 ***	0.152	0.006**	0.010**	0.359
	0.041*	0.001**	0.003**	0.001**	0.089˚	0.005**	0.009**	0.170
Female head of HH	0.995	1.020	1.006	1.017	0.963	0.973	0.992	0.987
	0.909	0.640	0.851	0.558	0.243	0.392	0.750	0.606
	0.358	0.252	0.338	0.241	0.129	0.182	0.320	0.241
Number of children under 5 in HH	1.106	1.130	1.074	1.091	1.021	1.026	1.008	1.014
	0.000 ***	0.000 ***	0.000 ***	0.000 ***	0.173	0.078˚	0.505	0.245
	0.001**	0.001**	0.001**	0.001**	0.096˚	0.049*	0.242	0.120
Caste (comp. to scheduled caste)								
Scheduled tribe	0.897		0.962		1.125		1.180	
	0.027*		0.279		0.002**		0.000 ***	
	0.020*		0.149		0.003**		0.001**	
Other backward caste	0.867		0.898		0.992		0.904	
	0.000 ***		0.000 ***		0.789		0.000 ***	
	0.001**		0.001**		0.320		0.001**	
Upper caste	0.671		0.752		0.913		0.873	
	0.000 ***		0.000 ***		0.014*		0.000 ***	
	0.001**		0.001**		0.012*		0.001**	
Religion (comp. to Hindu)								
Muslim	1.054		0.969		0.963		0.862	
	0.244		0.337		0.286		0.000 ***	
	0.129		0.175		0.151		0.001**	
Other (Sikh, Christian, Buddhist, Traditional…)	0.974		0.749		0.748		0.807	
	0.752		0.000 ***		0.000 ***		0.000 ***	
	0.320		0.001**		0.001**		0.001**	
Wealth quintile (comp. to lowest)								
Lower middle	0.865		0.893		0.943		0.976	
	0.000 ***		0.000 ***		0.056˚		0.362	
	0.001**		0.001**		0.038*		0.184	
Middle	0.816		0.780		0.893		0.974	
	0.000 ***		0.000 ***		0.001**		0.381	
	0.001**		0.001**		0.002**		0.193	
Upper middle	0.725		0.689		0.840		0.930	
	0.000 ***		0.000 ***		0.000 ***		0.027*	
	0.001**		0.001**		0.001**		0.020*	
Highest	0.639		0.575		0.763		0.887	
	0.000 ***		0.000 ***		0.000 ***		0.002**	
	0.001**		0.001**		0.001**		0.003**	
Season of interview (comp. to winter)								
Summer	1.002	0.973	1.204	1.166	1.224	1.215	1.189	1.197
	0.960	0.487	0.000 ***	0.000 ***	0.000 ***	0.000 ***	0.000 ***	0.000 ***
	0.369	0.222	0.001**	0.001**	0.001**	0.001**	0.001**	0.001**
Monsoon	1.145	1.127	1.460	1.395	1.426	1.393	1.295	1.306
	0.002**	0.004**	0.000 ***	0.000 ***	0.000 ***	0.000 ***	0.000 ***	0.000 ***
	0.003**	0.005**	0.001**	0.001**	0.001**	0.001**	0.001**	0.001**
Autumn	1.142	1.187	1.100	1.114	1.191	1.256	1.102	1.135
	0.157	0.045*	0.357	0.275	0.118	0.035*	0.351	0.190
	0.091˚	0.029*	0.183	0.129	0.072˚	0.023*	0.181	0.098˚
Private improved toilet	0.870	0.686	0.876	0.676	0.909	0.802	0.916	0.826
	0.000 ***	0.000 ***	0.000 ***	0.000 ***	0.000 ***	0.000 ***	0.000 ***	0.000 ***
	0.001**	0.001**	0.001**	0.001**	0.001**	0.001**	0.001**	0.001**
Communal characteristics								
District toilet prevalence	0.932	0.612	0.450	0.304	0.596	0.503	0.644	0.566
	0.452	0.000 ***	0.000 ***	0.000 ***	0.000 ***	0.000 ***	0.000 ***	0.000 ***
	0.220	0.001**	0.001**	0.001**	0.001**	0.001**	0.001**	0.001**
Urban	1.011		1.037		1.038		0.954	
	0.800		0.173		0.190		0.042*	
	0.320		0.096˚		0.104		0.029*	
Coastal	0.835		1.013		1.202		1.063	
	0.008**		0.842		0.006**		0.383	
	0.007**		0.336		0.006**		0.193	
ICC	0.102	0.104	0.045	0.041	0.043	0.040	0.062	0.059

Regressions were estimated in the same way as regressions in Table 2 but below adjusted odd ratios (AOR) next to variables, p values rather than standard errors are reported. Below the p values are False Discovery Rate sharpened q values estimated using Anderson’s (2008) code. The N for all regressions is 57,021

***p < 0.001; ***p* < 0.01; **p* < .05; °*p* < 0.1

**Table 9:** Links between IYCF and nutrition outcomes in 4- and 5-month-old children

Nutrition outcome	Stunted	Underweight	Wasted	Anaemic
Model				
Infant & young child feeding				
Weaned (fed semisolid food)	0.866	0.787**	0.961	1.051
	0.082	0.069	0.077	0.073
Breastfed	0.783	0.775	0.859	1.144
	0.157	0.146	0.153	0.179
Child characteristics				
Female child	0.763***	0.915	0.977	0.969
	0.050	0.055	0.056	0.049
5-month-old (comp. to 4-month-old)	1.044	1.081	1.019	0.890*
	0.069	0.065	0.059	0.045
Preterm	1.375***	1.431***	0.887	1.189˚
	0.164	0.157	0.100	0.119
Any prelacteal feed	0.996	0.933	0.905	1.097
	0.092	0.078	0.072	0.078
Breastfed within 1 h of birth	1.002	0.990	0.989	0.972
	0.080	0.071	0.068	0.060
Had diarrhoea in the last 2 weeks	0.835˚	1.046	1.046	1.001
	0.081	0.088	0.085	0.074
Birth order	1.012	1.032	1.010	1.054˚
	0.035	0.033	0.030	0.029
Birth interval	0.996	0.994***	0.999	0.998
	0.002**	0.001		0.001
Household characteristics				
Mother’s age at birth	0.990	1.003	1.013	0.972***
	0.010	0.009	0.008	0.007
Mother undernourished	1.125	1.669***	1.341***	1.338***
	0.105	0.137	0.109	0.104
Mother’s education in years	0.964***	0.978**	0.999	0.980**
	0.008	0.008	0.007	0.006
HH size	0.995	0.999	0.997	1.017
	0.014	0.012	0.012	0.011
Female head of HH	1.084	1.047	1.162˚	0.988
	0.109	0.097	0.101	0.078
Number of children under 5 in HH	0.976	1.009	1.111**	0.998
	0.045	0.043	0.045	0.036
Caste (comp. to scheduled caste)				
Scheduled tribe	1.175	0.862	0.751**	1.076
	0.142	0.090	0.076	0.101
Other backward caste	0.959	0.784**	0.884	0.826**
	0.088	0.065	0.070	0.060
Upper caste	0.865	0.667***	0.825*	0.721***
	0.102	0.070	0.081	0.063
Religion (comp. to Hindu)				
Muslim	0.930	0.903	0.981	
	0.101	0.087	0.088	0.880
Other (Sikh, Christian, Buddhist, Traditional…)	0.934	0.557***	0.729**	0.072
	0.162	0.076	0.089	0.715**
Wealth quintile (comp. to lowest)				0.076
Lower middle	0.875	0.955	1.004	0.939
	0.084	0.083	0.086	0.073
Middle	0.785*	0.837˚	1.026	0.816*
	0.087	0.083	0.098	0.070
Upper middle	0.673**	0.696**	0.912	0.854˚
	0.085	0.079	0.098	0.081
Highest	0.744*	0.818	1.014	0.715**
	0.107	0.105	0.122	0.076
Sanitation				
Private improved toilet	0.890	0.863˚	1.045	1.058
	0.076	0.066	0.076	0.068
District toilet prevalence	0.932	0.680˚	0.603**	0.555***
	0.292	0.137	0.110	0.097
Communal characteristics				
Urban	1.210*	1.167˚	1.006	0.942
	0.108	0.095	0.077	0.064
Coastal	0.875	0.803	1.022	1.097
	0.181	0.124	0.170	0.138
Interview time				
Season (comp. to winter)				
Summer	1.001	1.271*	1.410***	1.283**
	0.115	0.134	0.138	0.116
Monsoon	1.183	1.343*	1.280*	1.587***
	0.158	0.158	0.139	0.163
Autumn	0.364**	0.854	1.099	1.767*
	0.130	0.230	0.265	0.403
ICC	*0.048*	*0.063*	*0.041*	*0.072*

The numbers next to the variables are AORs, below are standard errors. The N for all regressions was 6,570. The regressions were estimated in the same way as those presented in Table 2

****p* < 0.001; ***p* < 0.01; **p* < .05; °*p* < 0.1

## Footnotes

1 Andaman and Nicobar Islands, Andhra Pradesh, Assam, Bihar, Dardra &Nagar Haveli and Daman & Diu, Goa, Gujarat, Himachal Pradesh, Jammu and Kashmir, Karnataka, Kerala, Ladakh, Lakshadweep, Maharashtra, Manipur, Meghalaya, Mizoram, Nagaland, Sikkim, Telangana, Tripura, and West Bengal.

2 About 26% of the Indian population is vegetarian (NFHS 2015–2016).

3 Although Dixit et al. (2018) again found the ICDS not to reduce stunting, wasting or underweight rates among beneficiary children.

4 The underlying rationale for doing so lies in previous research (Krasevec et al., 2017, p. 9) highlighting a link between the consumption of more types of ASF and lower stunting, either because such consumption could be an approximate measure for the quantity of ASF intake or because different ASF types may support growth in distinct ways, due to their varied nutrient profiles.

5 Calculated as district-level leave-out means.

6 There are some traditional beliefs in India about what types of foods can be eaten in different seasons that could in turn affect children’s nutrition status.

7 Because the wealth index quintiles calculated by the NFHS/DHS are based on data that include information about toilet and livestock ownership, both of which are investigated as separate variables in this study, I re-estimated the wealth index following the DHS guidelines (Rutstein, 2015) but without data on toilet and livestock ownership.

8 However, these significant results largely disappear among the 4- and 5-month-old cohort, as Table 9 in the Appendix examining the relationship between weaning and nutrition outcomes in this younger age group demonstrates.

9 For children under 1 year old, breastfeeding is associated, albeit not significantly, with lower malnutrition rates. The lack of significance may be driven by the fact that the vast majority of such young children in the sample are breastfed.

10 And subtraction of weaned and minimum dietary diversity.

11 Despite being linked with nutrition outcomes in some existing research, immunisation status and receipt of antenatal care did not demonstrate a significant relationship with children’s nutrition outcomes in any model in this analysis and therefore were eventually not included.

12 This could be at least partially attributed to traditional beliefs that animal-sourced food should not be consumed in hot seasons such as the summer and monsoon (Sabharwal, 2014).

13 Southern states (Andaman and Nicobar Islands, Andhra Pradesh, Karnataka, Kerala, Lakshadweep, Puducherry, Tamil Nadu, Telangana), North-Eastern states (Arunachal Pradesh, Assam, Manipur, Meghalaya, Mizoram, Nagaland, Sikkim, Tripura), Eastern states (Bihar, Jharkhand, Odisha, West Bengal), Northern states (Chandigarh, Haryana, Himachal Pradesh, Jammu and Kashmir, Delhi, Punjab, Rajasthan), Central states (Chhattisgarh, Madhya Pradesh, Uttar Pradesh, Uttarakhand), and Western states (Dardra and Nagar Haveli, Daman and Diu, Goa, Gujarat, Maharashtra).

14 88% boys as opposed to 87% girls.

15 The models were estimated utilising multilevel logistic regressions akin to those in Table 2. The effects of livestock ownership on children’s nutrition were estimated with the same models as those in Table 2, with the addition of the livestock variables. The models examining the effects of livestock ownership on the consumption of different food types were similar, with the omission of most IYCF variables except for breastfeeding.

16 Such disaggregation was not possible with the NFHS IV data.

17 In terms of Indian regions, the highest poultry-ownership prevalence is in the North-East (57%) and the lowest (4%) in the North. From Indian states, Nagaland has the highest (73%) and Haryana the lowest (2%) prevalence of poultry ownership.

18 More details about the PSM estimations can be found in the Appendix.

19 The diversification of India’s Public Distribution System, which allows poorer sections of the population to purchase subsidised staple foods, beyond grains and pulses to include also ASF, fruits, and vegetables could thus likely be very helpful here.

20 The fact that district-level poultry ownership is associated even more strongly and consistently with the consumption of ASF than household-level poultry ownership also provides support for this conjecture. The direction of the relationship is not clear from this research, however, i.e. whether the greater poultry ownership encourages greater ASF consumption or greater acceptance of ASF consumption leads to greater poultry ownership.

21 Greater government investment in poultry ownership could also be useful here but the link between poultry ownership, greater consumption of animal-sourced food, and nutrition outcomes is complex as I discuss in the next paragraph and would require more in-depth research to fully elucidate. Such research could also look further into how livestock ownership in different areas relates to prevalent norms about appropriate child feeding.

22 Government policy could aim to raise awareness in this regard as well, for example about the benefits of keeping livestock separate from the families’ living areas (Ngure et al., 2014).

## Code availability

Code will be shared through Figshare upon publication.
