# Supplementary material for: The role of complementary feeding in India’s high child malnutrition rates: findings from a comprehensive analysis of NFHS IV (2015–2016) data
Source: Food Secur. 2021 Sep 28;14(1):39–66. doi: 10.1007/s12571-021-01202-7 (PMC8477628; doi:10.1007/s12571-021-01202-7)

**Propensity score matching for estimating the role of ICDS services in children’s IYCF and nutrition outcomes**

To estimate the links between ICDS participation and beneficiary children’s feeding habits and nutrition outcomes, I used Propensity Score Matching (PSM), which allows for impact evaluation even in the absence of panel data (e.g. Dixit et al. 2018, Kandpal 2011, Ravallion 2001). This quasi-experimental approach is based on the construction of a suitable control group to the treated one (=households with children that received a certain service from the ICDS), whose outcome indicators are then subtracted from the treatment groups’ to determine the significance and size of the impact.

The validity of PSM rests on two assumptions. The first one, expressed as

*0<P(X)<1*

*where P(X) are the propensity scores calculated based on observable variables X*

implies that valid matches on P(X) can be found for all values of observable variables X. The second one can be arithmetically expressed as

*E(Yt0| X ,D = 1)= E(Yt0| X ,D = 0)*

and it presumes that conditional on X, households that did not receive the specific type of ICDS help examined have the same outcomes in the analysed variables as the households that receive the service would have had they not been participating.

Scholars (e.g. Rubin 1973 as cited in Kandpal 2011: 1413) showed that ‘PSM eliminates selection bias if controlling for X eliminates selection bias from endogenous placement.’ Nevertheless, given that not all potential determinants of ICDS participation on the household or child level are observable, the method helps reduce but cannot completely eliminate selection bias.

The specific methods of matching used were the nearest neighbour and five-nearest-neighbours approaches.

The treatment variables investigated included whether a child participates in all aspects of ICDS (receives ICDS food at least weekly, regularly attends ICDS preschool, and receives monthly weighing and check-ups) and whether s/he gets food from the programme at least weekly or daily. The outcome variables examined included the effects of the programme’s services on children’s IYCF (weaned, satisfactory dietary diversity, and consumption of any ASF and of vitamin-A-rich fruits and vegetables) and nutrition outcomes (stunted, underweight, wasted, and anaemic).

The first step in the matching was to calculate the propensity scores, i.e. observable factors that are likely to affect the participation in ICDS as well as the outcome variables. These included child-level variables (gender, age, born preterm, birth order, birth interval with preceding sibling, still breastfed, breastfed within one hour of birth, received vitamin-A supplementation in the last 6 months, had diarrhoea in the last two weeks), maternal and household characteristics (mother’s age, years of education completed, and her nutrition status, household size, whether a household is female-headed, household complete wealth index, religion, and caste) and communal characteristics (district-level prevalence of toilets, if a district is coastal, if an area is urban, and the region and state of residence). In case of propensity scores for ICDS food services, we also control for the other ICDS services that households may be receiving, to maximise the comparability of the treated and control groups. The estimation method used to calculate the propensity scores was akin to the one used with the principal models, via a multilevel logistic regression with the first level the individual/households, second level the district, and third level the Indian state of residence.

The graphs below graphically display the propensity scores of the treatment and control groups, for each of the ICDS services examined, before and after matching. Importantly, they show that for all three services, the majority of propensity scores of the treatment and control group overlap – aka, are ‘on support’. The graphs after matching also demonstrate the greater balance of the treated and control groups after matching for the propensity scores.


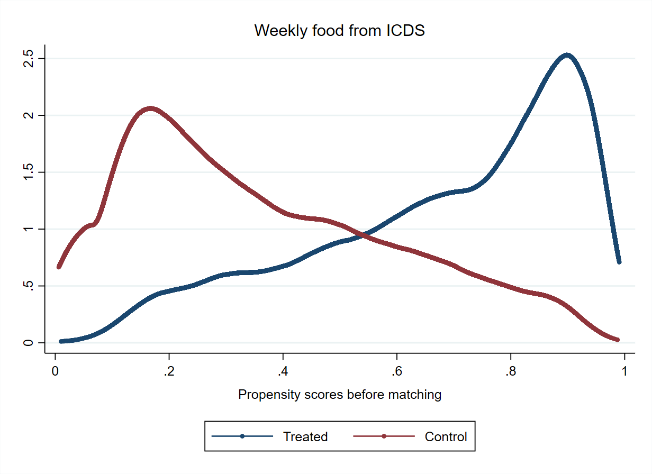

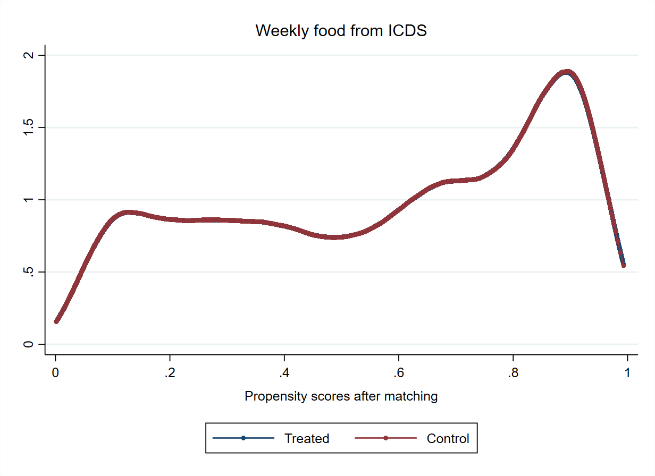


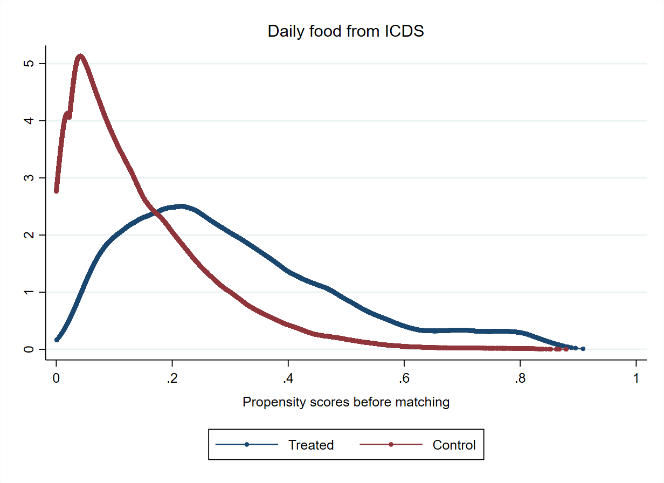

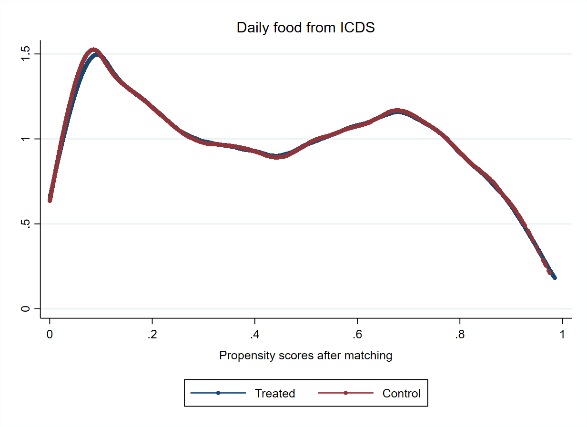


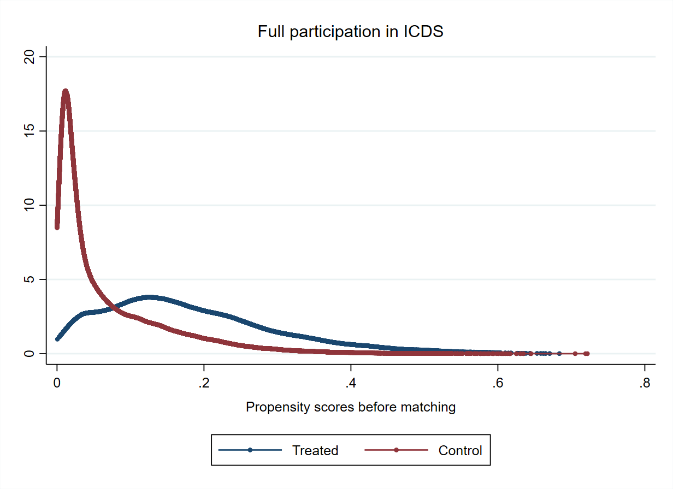

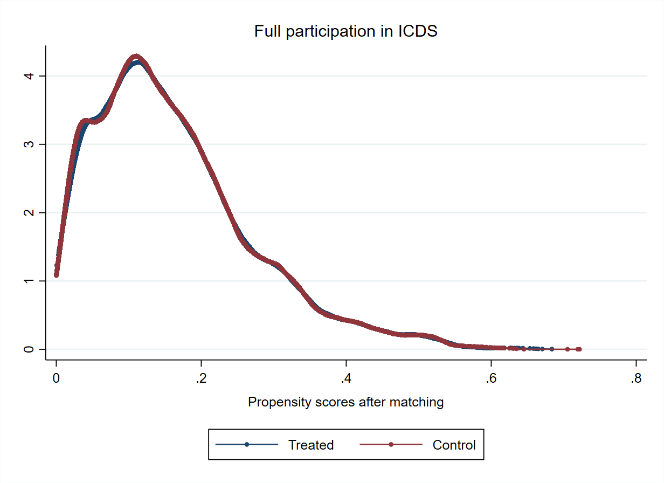

Supplement: Supplementary file 1 — Supplementary file1 (DOCX 313 kb) [file 12571_2021_1202_MOESM1_ESM.docx]
